# A novel, stain-free, natural auto-fluorescent signal, Sig M, identified from cytometric and transcriptomic analysis of infectivity of *Cryptosporidium hominis* and *Cryptosporidium parvum*

**DOI:** 10.3389/fcimb.2023.1178576

**Published:** 2023-05-22

**Authors:** Paul Ogbuigwe, Joanna M. Roberts, Matthew A. Knox, Axel Heiser, Anthony Pita, Neville A. Haack, Juan Carlos Garcia-Ramirez, Niluka Velathanthiri, Patrick J. Biggs, Nigel P. French, David T. S. Hayman

**Affiliations:** ^1^ School of Veterinary Science, Hopkirk Research Institute, Massey University, Palmerston North, New Zealand; ^2^ Flowjoanna Tāpui Ltd, Palmerston North, New Zealand; ^3^ Animal Health Solutions, Hopkirk Research Institute, AgResearch Ltd., Massey University, Palmerston North, New Zealand

**Keywords:** cryptosporidiosis, flow cytometry, spectral cytometry, intracellular infection, nanostring

## Abstract

Cryptosporidiosis is a worldwide diarrheal disease caused by the protozoan *Cryptosporidium*. The primary symptom is diarrhea, but patients may exhibit different symptoms based on the species of the *Cryptosporidium* parasite they are infected with. Furthermore, some genotypes within species are more transmissible and apparently virulent than others. The mechanisms underpinning these differences are not understood, and an effective *in vitro* system for *Cryptosporidium* culture would help advance our understanding of these differences. Using COLO-680N cells, we employed flow cytometry and microscopy along with the *C. parvum*-specific antibody Sporo-Glo™ to characterize infected cells 48 h following an infection with *C. parvum* or *C. hominis*. The *Cryptosporidium parvum*-infected cells showed higher levels of signal using Sporo-Glo™ than *C. hominis*-infected cells, which was likely because Sporo-Glo™ was generated against *C. parvum*. We found a subset of cells from infected cultures that expressed a novel, dose-dependent auto-fluorescent signal that was detectable across a range of wavelengths. The population of cells that expressed this signal increased proportionately to the multiplicity of infection. The spectral cytometry results confirmed that the signature of this subset of host cells closely matched that of oocysts present in the infectious ecosystem, pointing to a parasitic origin. Present in both *C. parvum* and *C. hominis* cultures, we named this Sig M, and due to its distinct profile in cells from both infections, it could be a better marker for assessing *Cryptosporidium* infection in COLO-680N cells than Sporo-Glo™. We also noted Sig M’s impact on Sporo-Glo™ detection as Sporo-Glo™ uses fluoroscein–isothiocynate, which is detected where Sig M also fluoresces. Lastly, we used NanoString nCounter^®^ analysis to investigate the transcriptomic landscape for the two *Cryptosporidium* species, assessing the gene expression of 144 host and parasite genes. Despite the host gene expression being at high levels, the levels of putative intracellular *Cryptosporidium* gene expression were low, with no significant difference from controls, which could be, in part, explained by the abundance of uninfected cells present as determined by both Sporo-Glo™ and Sig M analyses. This study shows for the first time that a natural auto-fluorescent signal, Sig M, linked to *Cryptosporidium* infection can be detected in infected host cells without any fluorescent labeling strategies and that the COLO-680N cell line and spectral cytometry could be useful tools to advance the understanding of *Cryptosporidium* infectivity.

## Introduction

Cryptosporidiosis is a globally ubiquitous disease caused by an infection with the parasite *Cryptosporidium* affecting humans, domestic animals, and wildlife. Human infection causes self-limiting diarrhea, usually lasting approximately 1 to 2 weeks post-infection (Su et al., 2019). However, the effects of the disease can be more severe in immunocompromised individuals and children under 5 years of age, and *Cryptosporidium* has been identified as the second most common cause of diarrhea in infants (Savioli et al., 2006). In 2011, it was estimated that approximately 83,000 children under 5 years old died globally due to cryptosporidiosis (Lanata et al., 2013). The real figure is probably higher due to the inefficient disease reporting mechanisms in some countries. Currently, nitazoxanide is the only drug approved by the Food and Drug Administration of the United States for the treatment of cryptosporidiosis (Manjunatha et al., 2016). However, this drug only partially alleviates the symptoms of the disease, and there are no effective vaccines.

Of the 38 currently accepted species of *Cryptosporidium*, *C. hominis* and *C. parvum* are the two types responsible for the most commonly reported infections in humans. The main mode of transmission is the fecal–oral route; however, recent evidence has shown that infection can be respiratory in humans and animals, invade the pancreatic and biliary systems, and, in rarer cases, lead to cerebral pathologies and cancer (Sponseller et al., 2014; Audebert et al., 2020; Gaber et al., 2020). The most common symptoms of infection are acute diarrhea and abdominal pain (Bones et al., 2019). However, symptoms such as nausea, vomiting, fever, nutrient malabsorption, and growth retardation (in children) have been reported in immunocompetent individuals (Tumwine et al., 2003; Feng and Xiao, 2017); severe malabsorption syndrome leading to mortality can occur in immunocompromised individuals (Chalmers and Katzer, 2013). Previous studies have found that the species of *Cryptosporidium* a person is infected with can influence the symptoms or sequelae that they experience—for example, eye pain and persistent headaches are symptoms associated with *C. hominis* infections but not with *C. parvum* (Hunter et al., 2004).

Evidence suggests that different genotypes within species can make different contributions in varied settings—for instance, the virulent *C. hominis* subtype family Ib is the main causative agent of cryptosporidiosis in high-income countries, such as Europe and North America, and is responsible for most outbreaks worldwide (Khan et al., 2018). In addition, the hyper-transmissible genotype of *C. parvum*, IIaA15G2R1, has been widely reported (Xiao, 2010). The waterborne mode of transmission of *Cryptosporidium* may cause population bottlenecks during dry seasons in places such as Bangladesh that could result in the selection of mutations that lead to an increase in infectivity of the parasite over time (Gilchrist, 2020).

Further understanding of the disease mechanisms of *Cryptosporidium*, which would allow us to make definitive associations between subtypes and infectivity, has been hampered by a lack of efficient *in vitro* systems (King et al., 2011; Peckova et al., 2016). Here infectivity is defined as the capacity of a pathogen to infect a susceptible host and complete its life cycle. The main cell lines that have been used for the culture of the parasite are the human colorectal adenocarcinoma cell line (Caco-2) and the human ileocecal colorectal adenocarcinoma cell line (HCT-8). While some success has been found in the complete and long-term cultivation of the parasite in these cell lines (Hijjawi et al., 2001; Winkworth et al., 2008; Tandel et al., 2019), other studies have limited success in recreating these results and achieved partial progression of the life cycle terminating in the asexual phase (Bones et al., 2019). For this reason, more complex experimental designs, such as the use of hollow fiber technology to augment cell culture and other organoids and bioengineered intestinal models, have been proposed and implemented (Morada et al., 2016; Gunasekera et al., 2020). Recently, Miller et al. (2018) adopted a new cell line, human esophageal squamous cell carcinoma (COLO-680N), which allows for the long-term cultivation of *Cryptosporidium* through its entire life cycle [but see Vélez et al. (2022)]. This is remarkable because most of the previously described cell lines used to culture *Cryptosporidium* are derived from intestinal cells. However, it is now understood that the parasite can infect multiple systems within the body (Jossé et al., 2019), so finding other suitable cell lines has the potential to benefit the field.

Taking advantage of advances in the culture of *Cryptosporidium*, the foundation of an *in vitro* system capable of assessing the infectivity of *C. parvum* and *C. hominis* is described here. The system uses the COLO-680N cell line to culture the parasite. Traditional or spectral flow cytometry in conjunction with the widely used *Cryptosporidium*-specific fluorescent reagent, Sporo-Glo™, is employed to assess the levels of infectivity from *Cryptosporidium* species. Sporo-Glo™ is a FITC-conjugated antibody targeting the intracellular life cycle stages of *C. parvum*. Flow cytometry is a laser-based method used to analyze the size and fluorescent characteristics of particles (Hui et al., 2022). Flow cytometers can identify small particles such as *Cryptosporidium* (King et al., 2009) using light scattering properties alone. The small particle size and correspondingly small natural auto-fluorescent signal produced by oocysts compared with mammalian cells have also been used to identify them (Sonzogni-Desautels et al., 2019). In general, traditional flow cytometry detects fluorescence from discreet portions of the fluorescent spectrum using band pass filters optimized for common fluorophores. Spectral flow cytometry detects fluorescence from the entire fluorescent spectrum, capturing signatures of light that are spectrally unmixed to identify particular fluorophore signatures. In this study, we pioneer using this approach to spectral detection to capture naturally arising, stain-free, full-spectrum fluorescent signatures from a number of players in the *in vitro* infectious ecosystem, including the parasite entities and host cells. *Cryptosporidium* infectivity in COLO-680N cells was also explored in a transcriptomic analysis, which was conducted to assess potential differences in infectivity between species. The NanoString nCounter^®^ analysis system hybridizes two probes (capture and reporter) with unique barcodes directly onto the RNA target without amplification, cDNA, or library preparation. This allows for the direct counting of each target molecule using an automated fluorescence microscope (Malkov et al., 2009; Eastel et al., 2019; Yu et al., 2019). NanoString, unlike RNAseq, does not necessitate the use of fold differences due to its ability to directly measure a broad range of mRNA expression levels without cDNA synthesis and amplification steps (Urrutia et al., 2016). We assessed the expression of *Cryptosporidium* and human host genes to attempt to gain a better understanding of the expression of genes at various stages of the life cycle of this pathogen. To that end, a panel of genes from previous transcriptomic studies (Lippuner et al., 2018; Matos et al., 2019) and a selection of potential drug targets (Manjunatha et al., 2017; Baragaña et al., 2019; Castellanos-Gonzalez et al., 2019; Su et al., 2019; Xu et al., 2019; Zhang et al., 2019; Mfeka et al., 2020) were chosen, and their expression was assessed.

## Methods

### Cryptosporidium samples

*Cryptosporidium parvum* and *C. hominis* oocysts were obtained from infected humans in New Zealand. The anonymized *Cryptosporidium*-positive fecal samples were sent to our laboratory from diagnostic laboratories across New Zealand under a Ministry of Health contract covering the time period between 2018 and 2021. All fecal samples were stored at 4°C before use. The species and the genotype of each sample were determined using PCR at the glycoprotein 60 (gp60) locus and subsequent Sanger sequencing.

### Purification of oocysts

*Cryptosporidium* oocysts were purified using modified methods from Meloni and Thompson (1996). Briefly, 0.5%, 1.0%, and 2% (w/v) Ficoll 400 (Merck KGaA, Darmstadt, Germany) solutions were prepared in phosphate-buffered saline (PBS) and stored at 4°C. Ficoll solution was layered using a pipette in a 2-ml safe-lock tube with the 2% solution at the bottom. Furthermore, 500 µl of oocyst solution was layered on top of the cold Ficoll gradient (4°C), and the tubes were centrifuged for 20 min at 1,500 × *g* at room temperature (RT). The interface was transferred, made up to 15 ml with PBS (4°C), and centrifuged for 5 min at 2,000 × *g* at 4°C. The supernatant was removed, leaving 1 ml of the PBS containing the purified oocysts which were transferred to a microcentrifuge tube with 15 µl of an antibiotic solution composed of 5 mg/ml gentamycin, 4 mg/ml lincomycin, and 10 mg/ml ampicillin before storage at 4°C.

### Excystation of oocysts and cell culture

Oocyst excystation was conducted by incubation of the oocysts at 37°C in an excystation solution composed of 0.8% taurocholic acid in PBS for 2 h (Petry and Harris, 1999).

COLO-680N cells (DSMZ Germany, ACC182) were maintained in 75-cm^2^ tissue culture flasks incubated at 37°C in a humidified incubator with 5% CO_2_ (Laurent et al., 1997; Miller et al., 2018) using a growth medium consisting of RPMI 1640 supplemented with 10% fetal bovine serum (FBS), 100 U/ml of penicillin, 100 µg/ml of streptomycin, and 250 ng/ml of amphotericin B (Jossé et al., 2019).

### *Cryptosporidium* infectivity assay

COLO-680N cells were seeded onto 12-well plates at 2.5 × 10^5^ cells/well. At 24 h later, *C. parvum* and *C. hominis* oocysts (containing four sporozoites) were excysted at different multiplicity of infection (MOI) values, where an MOI of 40 is equivalent to 2.5 × 10^6^ excysted oocysts/well. The excysted oocysts were centrifuged at 13,200 × *g* for 3 min and resuspended in the growth medium. Furthermore, 100 µl of the excysted oocyst suspension, adjusted to achieve the correct MOI, was spiked into each well, and the plates were spun at 188 × *g* for 7 min to encourage invasion and then incubated for 48 h. The growth medium was removed from each well, and then the wells were washed twice with 1 ml of PBS (Thermo Fisher Scientific, Waltham, MA, USA) to remove the excess sporozoites, oocysts, and oocyst shells. Cells were harvested using 300 µl of 0.25% trypsin-EDTA per well for 13 min at 37°C. Following the inactivation of 0.25% trypsin-EDTA with growth media, cells were harvested and spun for 3 min at 400 × *g* at RT. The pellet was resuspended in 1 ml of PBS. In some experiments, the samples were stained with eBioscience™ Fixable Viability Dye eFluor™ 780 (FV780) (Thermo Fisher Scientific, Waltham, MA, USA) for 30 min at 4°C in the dark, followed by two washes. The samples were washed and resuspended in 500 µl of 0.22-µm filter-sterilized FluoroFix™ Buffer (BioLegend, San Diego, CA, USA) for 30 min in the dark at RT. We assessed this fixation regime to confirm that *Cryptosporidium* used in this study was rendered non-viable through this approach so that the samples could be handled on the bench at the cytometer with reduced biohazard risk (data not shown). Following washing in PBS, the samples were stored at 4°C in PBS and protected from light before further manipulation and analysis, which usually occurred within 24 h. Replicate cultures for each condition were typically prepared, either in duplicate or triplicate. The data points plotted for the flow cytometry results represent individual measurements from separate independent cultures.

### *Salmonella* Typhimurium culture and infectivity assay

*Salmonella* Typhimurium (*Salmonella enterica* serovar Typhimurium) was cultured according to Abernathy et al. (2013). Briefly, *S.* Typhimurium grown at 37°C overnight in Luria–Bertani (LB) broth was sub-cultured for 3 h in pre-warmed (37°C) LB broth before the challenge to ensure log-phase cultures. COLO-680N cells were challenged with *S.* Typhimurium at an MOI of 100 by replacing the media with *Salmonella* infectious media. Immediately upon challenge, the plates were centrifuged at 500 × *g* for 5min, placed in a CO_2_ incubator, and allowed to incubate for 3 h before harvest.

### Sporo-Glo™ flow cytometry

The fixed samples were washed twice in filtered intracellular permeabilization buffer (IPB) (BioLegend, San Diego, CA, USA), taking care to disrupt the pellet between each wash, and the pellet was resuspended in 500 µl of 0.22 µm filter-sterilized blocking buffer (5% FBS in PBS) and incubated for 30 min in the dark at RT. The samples were washed twice in IPB and resuspended in 200 µl of 1:16 Sporo-Glo™ (Waterborne Inc., New Orleans, LA, USA) for 1 h in the dark at RT before being washed twice and resuspended in 400 µl PBS for data acquisition. When assessing the Sporo-Glo™ on COLO-680N cells, Sporo-Glo™-stained but uninfected cells were used as a negative control, Sporo-Glo™-stained sporozoites were included as a positive stain control to confirm the success of Sporo-Glo™ staining, and heat-shocked (dead) cells were included as a FV780 positive stain control. Samples of PBS, IPB, and blocking buffer were included as buffer controls. The samples were measured using a FACSVerse™ flow cytometer (BD biosciences, San Jose, CA, USA) (traditional flow cytometer) and a three laser (3L) Cytek^®^ Aurora (Cytek biosciences, Fremont, CA, USA) (spectral cytometer). Data from the Aurora were analyzed using Spectroflo^®^ (Cytek biosciences, Fremont, CA, USA) and FlowJo^®^ (BD biosciences, San Jose, CA, USA) software. Data from the FACSVerse™ were analyzed using FlowJo^®^ software.

### Sig M flow cytometry

When assessing Sig M using traditional flow cytometry, unstained but infected cultures were used to identify highly fluorescent host cells across a range of detectors. Uninfected cells and oocysts were used as controls. When assessing Sig M using spectral cytometry, the subset of cells from the highest MOI for *C. parvum* or *C hominis* infections that were brightly fluorescent in the V7 detector (Cytek 3L Aurora) were defined as the positive control for spectral unmixing, while cells from uninfected cultures were defined as the negative control for spectral unmixing. Autofluorescence extraction was included in the Spectroflo software unmixing workflow as this improved the resolution of Sig M (data not shown). *S.* Typhimurium-infected cells were measured in experiments with spectral unmixing using the described controls mentioned above in order to probe for the presence of Sig M in off-target intracellular infections. The samples were measured, and the data were analyzed using the instruments and software packages mentioned above.

### *Cryptosporidium* infectivity assay for microscopy

Clean cover slips were placed in the wells of six-well tissue culture plates, and seeded in the center of each well were 1 × 10^6^ COLO-680N cells (70% confluency). Then, 2 ml of growth medium was added and, at 24 h later, inoculated with excysted oocysts. The inoculated plates were spun down at 188 × *g* for 7 min at 4°C and incubated at 37°C in a humidified incubator with 5% CO_2_ for 48 h. The media was removed, followed by three washes in 1 ml PBS. The cells were fixed by adding 1 ml of FluoroFix™ buffer to each well and incubating for 30 min in the dark at RT. The cells were washed once with PBS and twice with IPB and then incubated for 30 min with 1 ml of blocking buffer in the dark. The cells were washed twice with IPB then Sporo-Glo™ at stock concentration, and 16× dilution was added to the relevant wells. The plates were incubated for 1 h in the dark at RT. The supernatant was removed, and the wells were washed twice in PBS before staining with 4′,6-diamidino-2-phenylindole (DAPI). The samples were washed (PBS), and the cover slip was removed prior to mounting and image capture using fluorescence microscopy.

### Assessment of IL-8 immune response from COLO-680N cells

COLO-680N cells were grown to >70% confluency in eight-well chamber slides (Thermo Fisher Scientific, Waltham, MA, USA), and the wells were inoculated with 4.25 × 10^5^ C*. parvum* sporozoites each or left untreated as controls. At 12 h later, 3 µg/ml Brefeldin A (Invitrogen, Carlsbad, CA, USA) was added, and the wells were incubated for 12 h more before staining. The wells were washed with PBS, and the cells were fixed by adding 2% paraformaldehyde (PFA) in PBS to each well for 20 min at RT. The PFA was removed, and 150 µl of 0.1% Triton^®^ X-100 (Invitrogen, Carlsbad, CA, USA) was added to each well and incubated for 10 min at RT. The wells were washed with 300 µl of 1% bovine serum albumin (BSA) blocking solution. The wells were stained with 60 µl DAPI (2 mg/ml), incubated for 30 min at RT in the dark, then washed twice with 300 µl BSA, and stained with 100 µl of Anti-Human IL-8 APC (eBioscience, San Diego, CA, USA) for 45 min at RT in the dark. After washing, the wells were mounted and imaged by fluorescent microscopy, and the images were processed using ImageJ software (Schneider et al., 2012).

### RNA isolation

Samples were collected at 24, 48, 96, and 120 h post-inoculation. Following incubation, excess sporozoites and oocysts were removed from each well by washing in 500 µl of PBS. The cells were harvested at all time points specified above using 0.25% trypsin-EDTA and resuspended in 500 µl of PBS prior to RNA extraction. An RNeasy mini kit (Qiagen, Hilden, Germany) was used for RNA extraction, and the protocol was executed according to the manufacturer’s instructions. The quantity of RNA in each sample was measured using a NanoDrop™ 2000 spectrophotometer (Thermo Fisher Scientific, Waltham, MA, USA); then, each sample was diluted where necessary by using a CentriVap^®^ (Labconco, Kansas City, MO, USA) complete vacuum concentrator to consolidate the RNA and then resuspending in RNAse-free water to a maximum RNA concentration of 128.5 ng/µl. The samples were stored at -80°C prior to sample preparation and NanoString™ (NanoString, Seattle, WA, USA) analysis.

### mRNA detection using nCounter^®^

mRNA detection was conducted using the nCounter^®^ (NanoString™) platform. The panel used consisted of 144 genes: 40 human genes frequently expressed in human cell lines selected as control genes to ascertain if the assay was functioning as expected and 104 *Cryptosporidium* genes extracted from the analysis of previous RNA-seq studies, with 48 thought to be expressed intracellularly and 48 extracellularly according to data from Matos et al., 2019 (**Supplementary Tables S2**, **S3**) and cross-referenced with Lippuner et al. (2018) (**Supplementary Table S4**.). The extra eight *Cryptosporidium* genes were selected based on the analysis of potential drug targets from recent studies (Manjunatha et al., 2017; Baragaña et al., 2019; Castellanos-Gonzalez et al., 2019; Su et al., 2019; Xu et al., 2019; Zhang et al., 2019; Mfeka et al., 2020). The gene IDs from the study were further cross-referenced with data from the CryptoDB database (http://cryptodb.org), from which the corresponding mRNA sequences were extracted. A full list of the gene IDs included in this study can be found in **Supplementary Table S1**.

Gene expression analysis was performed using the nCounter^®^ Analysis System (NanoString Technologies Inc., Seattle, WA, USA). The RNA samples were thawed on ice. The samples were hybridized by adding 8 μl of MasterMix and 7 μl of RNA per tube of a 12-tube strip immediately before placing the strip at 67°C for 22 h. After hybridization, the samples were transferred to the nCounter^®^ Prep Station which automatically removed the excess probe and aligned and immobilized the probe–target complexes in the nCounter^®^ cartridge. The sample cartridges were placed in the nCounter^®^ Digital Analyzer which counted and tabulated color codes on the surface of the cartridge for each target molecule. Data were retrieved from the Analyzer as raw data (Reporter Code Count, RCC) files.

### Gene expression data analysis

The raw reporter code counts were retrieved from the analyzer in a tabulated data file (RCC) and imported into the nSolver Analysis software, version 4.0 (https://www.nanostring.com/products/analysis-solutions/ncounter-analysis-solutions/) for analysis. A reporter library file specific to our 144-gene CodeSet was provided by the manufacturer; it contained information such as the assignment of probe to gene. This file was used by the nSolver software to execute its quality control (QC) program on the samples using these parameters: fields of view registration <75% (imaging QC), binding density outside of 0.1–2.25 range (binding density QC), positive control *R* ^2^ value <0.95 (positive control linearity QC), and 0.5 fM positive control ≤2 SD above the mean of negative controls (positive control limit of detection QC). All samples passed the QC; however, there was a limit of detection QC flags present in multiple samples due to a low level of detection of *Cryptosporidium*-specific genes (see “Results”). Positive controls (spiked by the NanoString Company in the CodeSet) were used for correcting the assay efficiency. Negative controls were used to filter out microRNAs with expression at noise level. Median normalization was performed to normalize across samples using all housekeeping genes, and heat maps were used for data visualization according to established protocols (Yu et al., 2019).

## Results

### Characterization of *Cryptosporidium* infection using Sporo-Glo™

**Figure 1:**
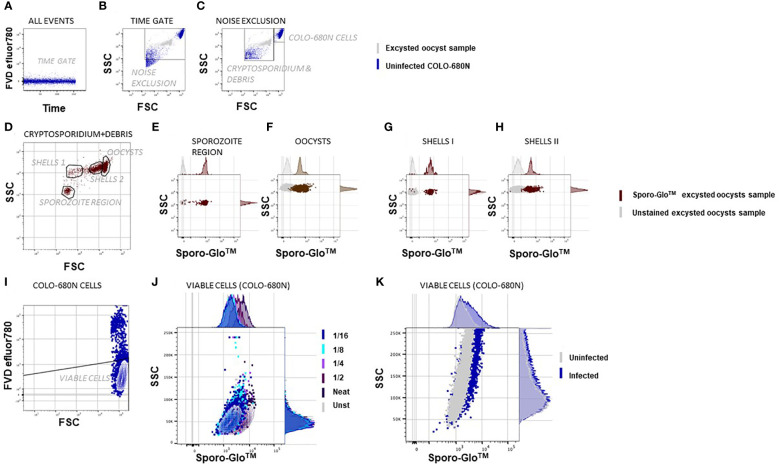
Flow cytometric *Cryptosporidium* infectivity assay detects infected COLO-680N cells at the same time as oocysts, sporozoites, and shells from *Cryptosporidium* using anti-Spor FITC. The samples were fixed, permeabilized, and stained with Sporo-Glo™ FITC as indicated. The population shown on the graph is indicated above each dot plot, and the populations/regions defined on dot plots are shown and labeled within the dot plot/contour plot. **(A)** A time gate verifies stream stability. **(B)** Excysted oocyst sample in gray and uninfected COLO-680N sample in blue overlaid to show the noise exclusion gate, which removes the debris of smaller side scatter signals than the smallest *Cryptosporidium* particles. **(C)** A region that captures COLO-680N cells is distinct from a region that captures *Cryptosporidium* and debris. **(D)** Four populations are evident based on unique FSC and SSC signals within the *Cryptosporidium* and debris region. **(E)** Particles in the sporozoite region stain clearly with Sporo-Glo™ FITC at 1/16 dilution. **(F)** Oocysts stain with the same concentration of anti-Spor FITC. **(G, H)** Two regions defined as shells—or empty oocysts—stain with anti-Spor FITC. **(I)** Viable COLO-680N cells are defined using FVD efluor780. **(J)** A titration of anti-Spor FITC on uninfected COLO-680N cells to capture the dilution with minimal background staining shows that 1/16 gives a similar signal intensity to unstained cells. **(K)** Infected COLO-680N cells stain with anti-Spor FITC.

COLO-680N cultures were infected with excysted *C. parvum* or *C. hominis* and assessed by using traditional flow cytometry (**Figure 1**). COLO-680N cells are relatively large and granular and can be easily identified at the top end of the light scatter dynamic range. Examining first uninfected COLO-680N cultures, Sporo-Glo™ (a commercially available polyclonal antibody preparation that is specific for *C. parvum* sporozoites) was titrated to reduce non-specific background staining on these cells (**Figure 1J**), and then infected cultures were evaluated using the chosen concentration of Sporo-Glo™. The cultures from this particular infection showed a small increase in Sporo-Glo™ signal on COLO-680N cells (**Figure 1K**).

Conserving the same cytometer settings, it was also possible to resolve *Cryptosporidium* oocysts, ghosts (non-acid-fast oocysts, potentially non-viable, empty oocyst walls), and sporozoites with a smaller relative light scatter compared with COLO-680N cells as expected. We used samples of intact or excysted oocysts alone to confirm that we could identify these particles (**Figure 1D**) independently of COLO-680N cells, and this allowed us to investigate and count free sporozoites or oocysts in infected COLO-680N cultures (**Supplementary Figure S1**). We observed that sporozoites could be detected near the lower limit of detection of light scatter, and while the particles in this region of the forward scatter (FSC) *vs*. side scatter (SSC) dot plot were not exclusively sporozoites, a distinct population of particles was resolved from the background with the inclusion of Sporo-Glo™ at the optimized concentration (**Figure 1E**). Sporo-Glo™ staining also revealed a positive FITC signal on oocysts and shells (**Figures 1F–H**).

**Figure 2:**
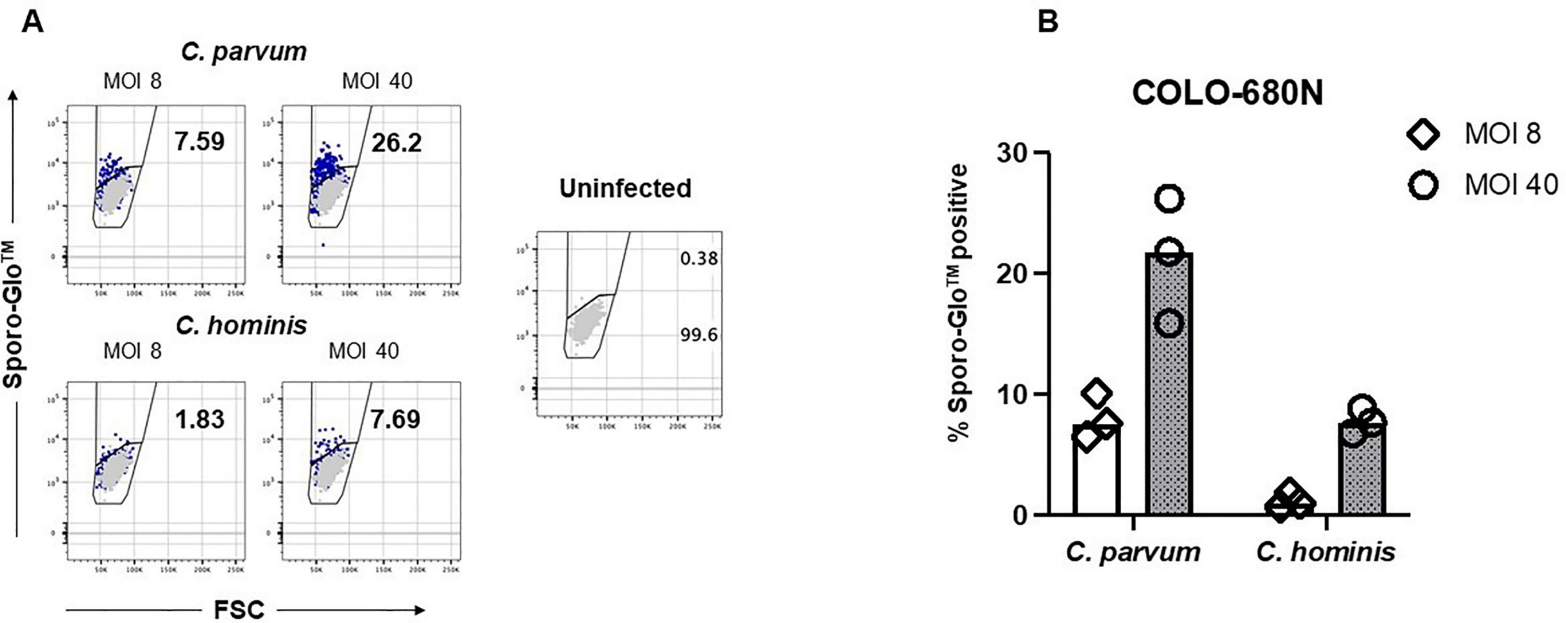
Sporo-Glo™-positive COLO-680N cells are detectable in increasing proportions with increasing multiplicity of infection (MOI) for *C. parvum* and *C. hominis*. **(A)** COLO-680N cells were infected at the indicated MOI (dark blue dots) with either *C. parvum* (top two panels) or *C. hominis* (bottom two panels) and then fixed, permeabilized, and stained with Sporo-Glo™. Uninfected cells stained with Sporo-Glo™ (light gray dots) are overlaid on each panel. A region defining Sporo-Glo™-positive cells is shown, and the percentage of cells in each region is displayed. **(B)** Triplicates from independent cultures from the experiment shown in **(A)** plotted as percent Sporo-Glo™ cells.

We reasoned that more Sporo-Glo™-positive COLO-680N cells might be expected to be found in samples from higher *Cryptosporidium* MOI when assessed by flow cytometry. **Figure 2** shows that a higher MOI does lead to a higher proportion of Sporo-Glo™-positive cells from infected cultures for both *C. parvum* and *C. hominis* infection. In this experiment, *C. parvum* infection produced the highest percentage of Sporo-Glo™-positive cells at a MOI of 40 (average 21.30%, *n* = 3), while an MOI of 8 gave an average percentage of Sporo-Glo™-positive cells of 8.07% (*n* = 3). This lower percentage of positive cells from MOI 8 for *C. parvum* was similar to the top value achieved at MOI 40 for *C. hominis* (7.78%, *n* = 3), while MOI 8 for *C. hominis* yielded very few positive cells. Several independent repeats of this experiment with the same and different MOIs show a similar trend (**Supplementary Figures S2**-**S4**).

Taking advantage of the ability of the FACSVerse cytometer to store a volume reading during measurement, we used this, along with the data analysis regime displayed in **Figure 1**, to estimate the absolute counts of oocysts and sporozoites present in infected cultures (**Supplementary Figure S1**). *C hominis* cultures had higher numbers of oocysts and sporozoites than C parvum cultures despite having few Sporo-Glo™-positive host cells (**Figure 2**).

**Figure 3:**
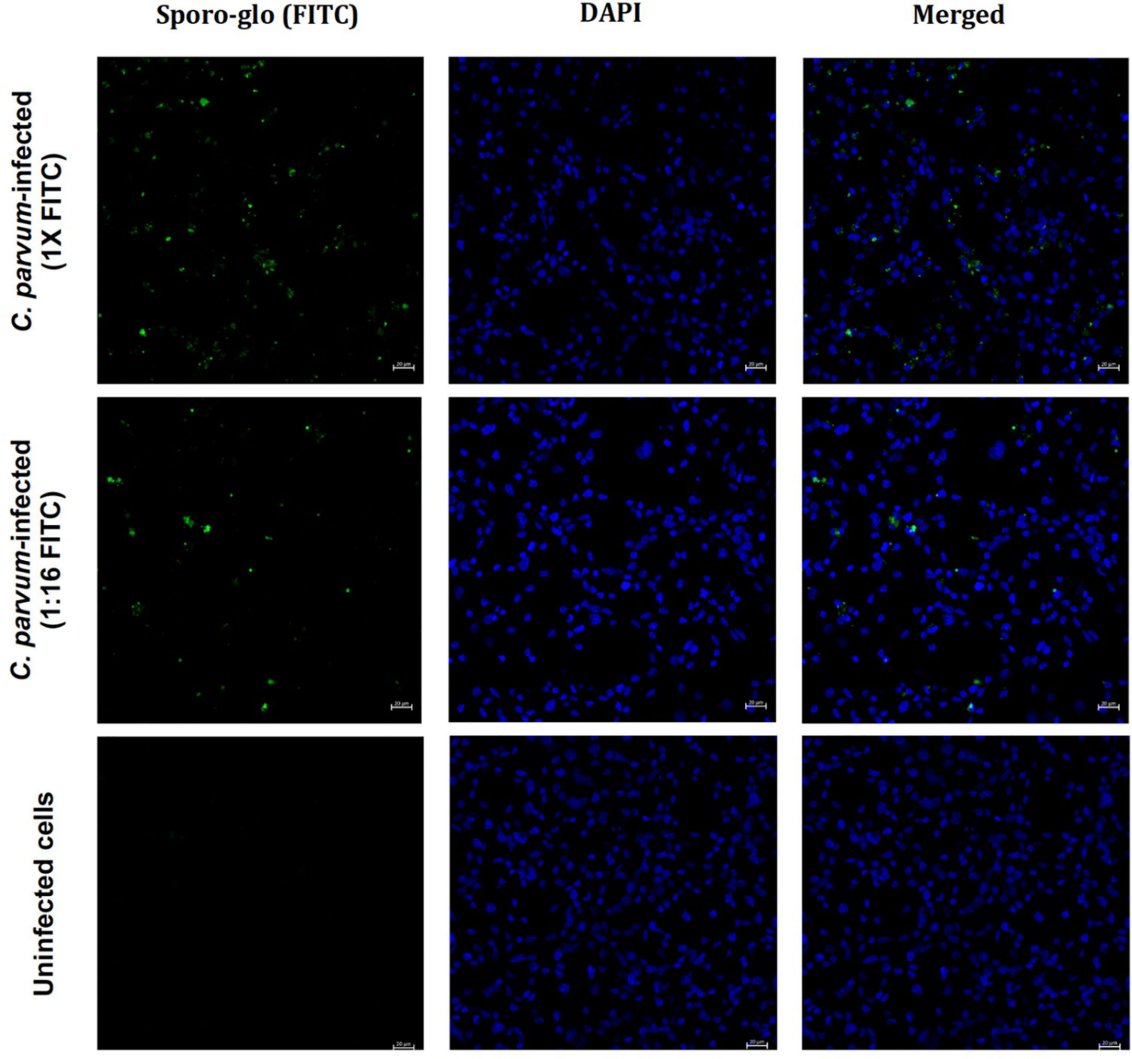
Detection of *C. parvum* in infected COLO-680N cells at 48 h post-inoculation. Cells were infected with *C. parvum* at a multiplicity of infection of 30. C*. parvum* was stained using Sporo-Glo™ (FITC) and is shown in green. The cell nuclei were stained with DAPI and are shown in blue. The scale bar represents 20 μm.

With a fluorescent microscope we assessed the Sporo-Glo™ signal in infected COLO-680N cell cultures cultivated in a six-well plate to allow direct staining and imaging *in situ* (**Figure 3**). Using *C. parvum* at an MOI of 30, **Figure 3B** shows a subset of cells from the infected cultures stained with Sporo-Glo™ at the optimized concentration used for flow cytometry. The uninfected cultures showed no background staining (**Figure 3C**). The *Cryptosporidium* signal is detectable away from the nucleus, which likely corresponds with the plasma membrane location of the infection. Furthermore, cells from infected cultures showed IL-8 secretion as established by using fluorescence microscopy (**Supplementary Figure S5**).

**Figures 1**–**3** and **Supplementary Figures S2**-**S4** together show that Sporo-Glo™ produces a signal that appears to be specific for *Cryptosporidium* particles (shells, oocysts, and sporozoites) when measured by using flow cytometry (**Figures 1E-H**) while only producing a low background signal on uninfected COLO-680N cells (**Figure 1J**). When Sporo-Glo™ is used to stain infected COLO-680N cultures, a sub-population of FITC positive cells is detected (**Figures 1K**, **2**) with both *Cryptosporidium* species. The presence of increasing proportions of these FITC positive cells as MOI increases points to the presence of a sub-population of infected cells that become more abundant at higher MOI (**Figure 2**), which can be detected by employing fluorescent microscopy using the same titer of reagent following the *C. parvum* infection (**Figure 3**).

### Sig M: a novel natural auto-fluorescent signature in *Cryptosporidium* infection

**Figure 4:**
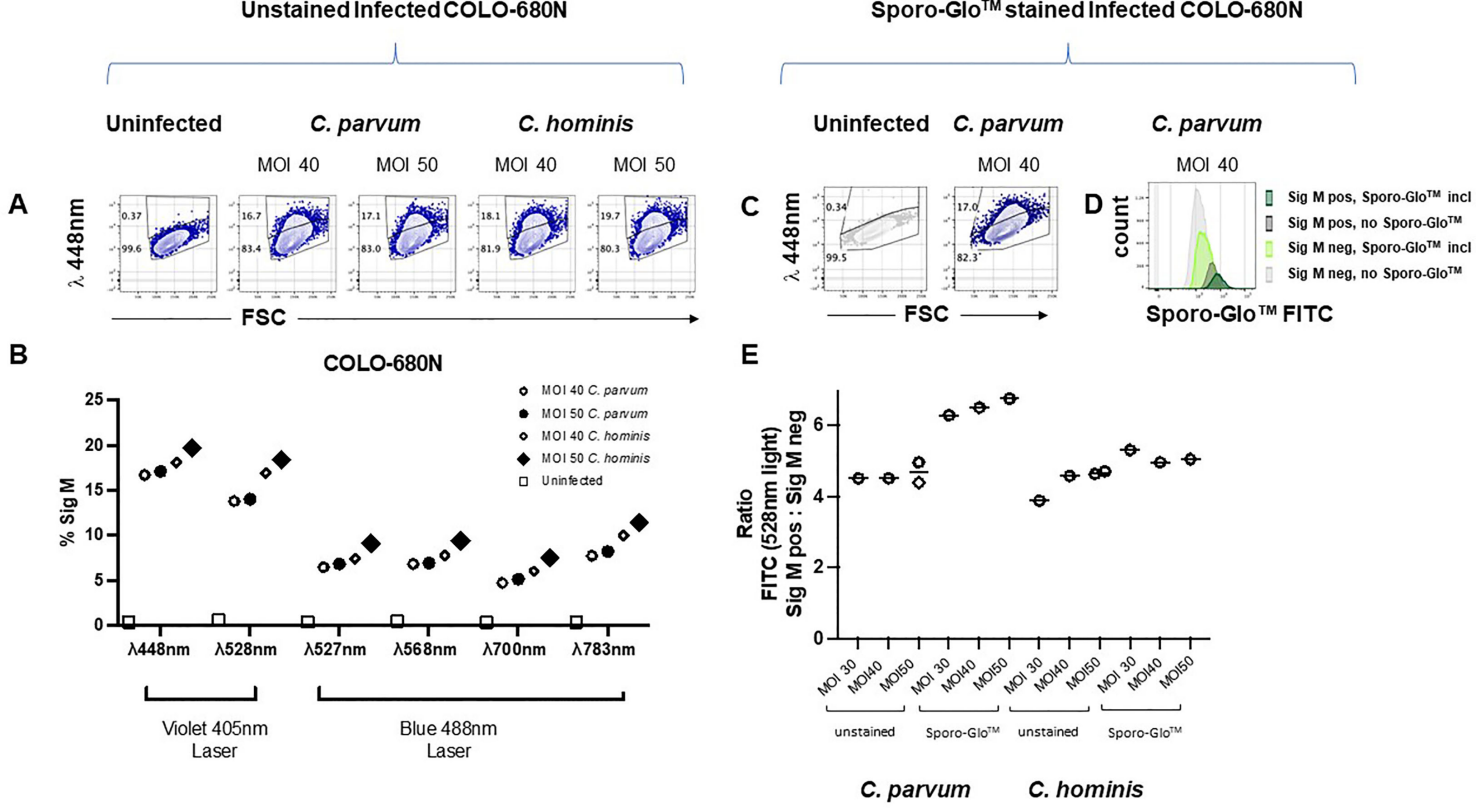
Fixed but completely unstained COLO-680N cultures infected with *C. parvum* and *C. hominis* contain a population of cells with a naturally auto-fluorescent profile (Sig M) that is absent from uninfected cultures. For each infection condition indicated, Sig M-positive cells are detected in infected cultures using a 448/45 BP filter with 405-nm excitation **(A)**. Percent of Sig M-positive cells from infected and uninfected completely unstained cultures captured with a range of fluorescent filters (448/45 BP filter with 405-nm excitation, 528/45 BP filter with 405-nm excitation, 527/32 BP filter with 488-nm excitation, 568/42 BP filter with 488-nm excitation, 700/54 BP with 488-nm excitation, and 783/56 BP filter with 488-nm excitation) show that Sig M is detectable across a broad range of wavelengths **(B)**. Fixed, permeabilized, and fully stained COLO-680N cultures (Sporo-Glo™ FITC, FVDefluor780) contain a population of Sig M-positive cells identifiable with 448/45 BP filter with 405-nm excitation from infected cultures—uninfected and *C. parvum* MOI 40 shown **(C)**. Comparing the fully stained and unstained infected cultures for FITC signal shows that Sporo-Glo™ staining increases the strength of FITC signal when compared with unstained infected cultures—*C. parvum* MOI 40 shown **(D)**. The ratio of FITC/lambda 528-nm light for Sig M high cells *vs*. Sig M low cells demonstrates a small increase in favor of a specific Sporo-Glo™ FITC signal for *C. parvum*-infected cultures, while the *C. hominis*-infected cultures may show a similar ratio regardless of Sporo-Glo™ staining **(E)**.

We coincidentally observed an unexpected increase in the autofluorescence of a subset of cells from infected COLO-680N cells when examining Sporo-Glo™ signals by using flow cytometry. This increased auto-fluorescence was detectable across a range of fluorescent filters and detectors on a traditional flow cytometer (BD FACSVerse) (**Supplementary Figure S6**). The proportion of cells with this increase in autofluorescence varied in proportion with the MOI. To confirm that this subset of auto-fluorescent cells was not related to an artefact of Sporo-Glo™ staining or FV780 (viability dye), a set of infected COLO-680N cultures was examined without Sporo-Glo™ staining. We found that infected but unstained COLO-680N cultures contain a sub-population of cells with enhanced autofluorescence compared with uninfected cultures (**Figures 4A, B**). This increased autofluorescence is detected across a range of wavelengths and from a number of laser excitations (**Figure 4B**), but the subset of cells with this profile was most abundant when measured using 405-nm laser excitation with collection at 448-nm wavelength (**Figure 4A**). We christened this auto-fluorescent signal Sig M. Surprisingly, the detector for Sporo-Glo™ (488-nm laser excitation, collection at 527-nm wavelength) was impacted by Sig M with a noticeable increase in autofluorescence in the presence of infection but with the absence of staining (**Figure 4C**). **Figure 4D** shows that the inclusion of Sporo-Glo™ to infected cultures produces a stronger signal than from Sig M alone in the absence of Sporo-Glo™ staining. Nevertheless, the uninfected cultures have a low level of Sporo-Glo™ signal when compared with the unstained uninfected cultures (**Figure 4D**).

A low level of Sporo-Glo™ background signal on uninfected cultures could suggest that the augmented Sporo-Glo™ detected on the stained sub-population of cells in infected cultures may just be the additive effect of Sig M and non-specific Sporo-Glo™ background. To normalize for low-level background staining from Sporo-Glo™ so that true Sporo-Glo™ signal could be assessed, we used unstained infected cultures to calculate the ratio of signal intensity in the FITC detector between Sig M-positive and Sig M-negative cells and compared this with the same ratio in Sporo-Glo™-stained infected cultures. We reasoned that, in the no-Sporo-Glo™ samples, this value would be constant across various MOI from within an experiment. On the other hand, higher ratios compared with these should be expected for infected cultures stained with Sporo-Glo™ if indeed Sporo-Glo™ is staining the positive cells specifically. **Figure 4E** shows that the ratio of light in the FITC detector from Sig M bright and dim populations in unstained cultures (in this case, “FITC” signal is only contributed by Sig M) is constant across various MOI for *C. parvum* and *C. hominis* as expected. Meanwhile the addition of Sporo-Glo™ leads to a higher ratio for *C. parvum*-infected cultures (**Figure 4E** and **Supplementary Figures S7**, **S8**), suggesting that both Sporo-Glo™ and Sig M are contributing to this measurement in the positive cells. In *C. hominis*-infected cultures, the picture is far less clear with only a modest or even no increase in this ratio (**Figure 4E** and **Supplementary Figures S7**, **S8**). These results suggest that Sporo-Glo™ is contributing a specific signal to the detection of *C. parvum*-infected COLO-680N cells but may not be contributing a specific signal to *C. hominis*-infected COLO-680N cells. The newly characterized signal, Sig M, is present on a clear subset of cells in infected cultures from both *C. hominis* and *C. parvum* infections and is detectable across a broad range of wavelengths (**Figure 4B** and **Supplementary Figure S6**). These results together suggest that Sig M and Sporo-Glo™ contribute to the positive cells detected in infected *C. parvum* COLO-680N cells, while *C. hominis*-infected COLO-680N cells contain a Sig M-positive population with limited contribution from Sporo-Glo™.

**Figure 5:**
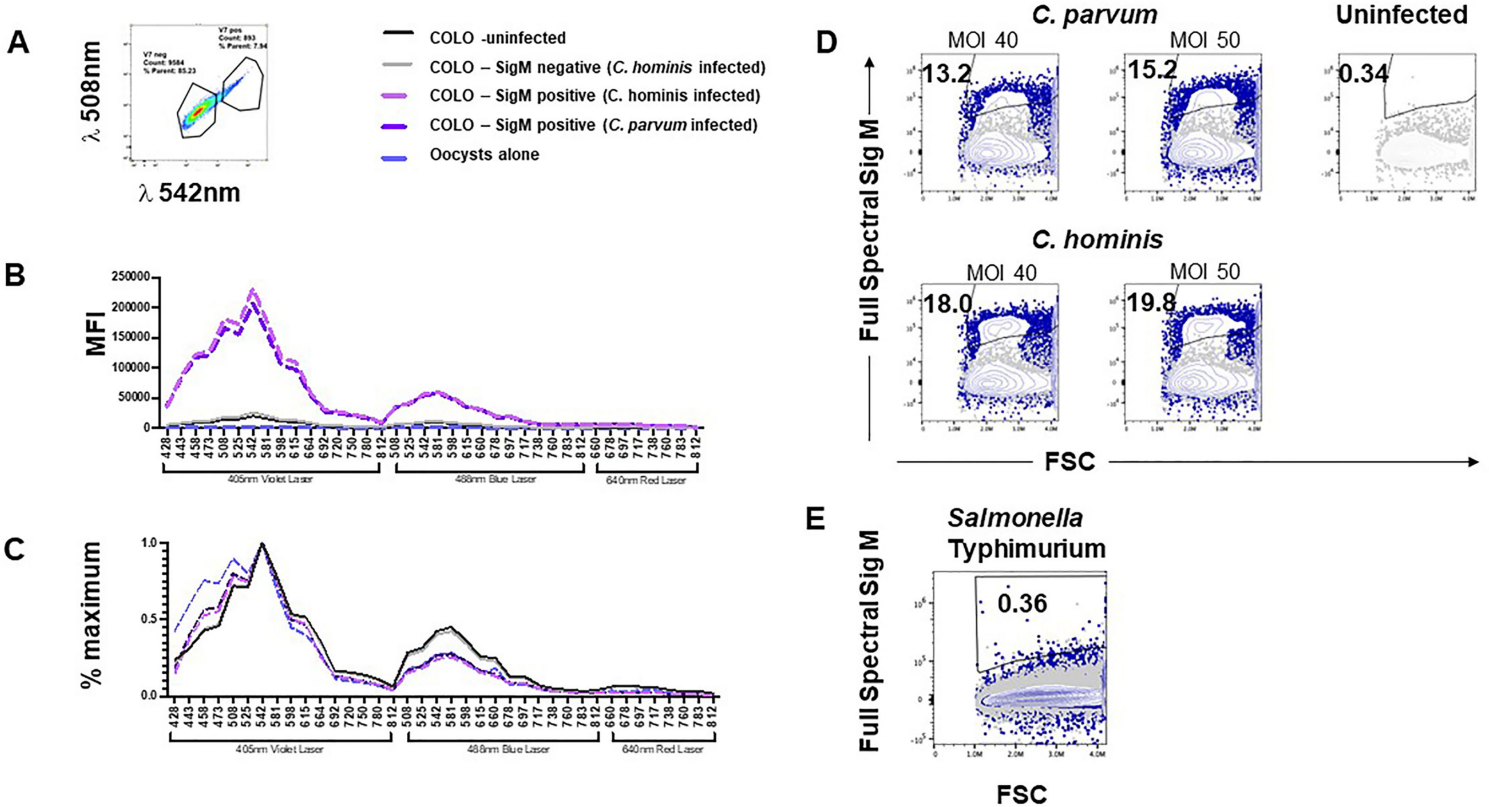
*Cryptosporidium*-infected, fixed, and unstained COLO-680N cultures measured using a spectral cytometer contain a population of cells distinguishable from uninfected COLO-680N cultures and not present in *Salmonella*-infected COLO-680N cultures. Selecting the brightest signal from COLO-680N cells from infected cultures (Sig M) using detector V7 (central wavelength: 542 nm) **(A)** allows the spectral profile of those cells to be plotted (media fluorescence intensity; MFI) across all detectors on a three-laser Cytekbio Aurora spectral cytometer **(B)**, revealing a very bright signal from these cells when compared with uninfected cultures and Sig M-negative cells from infected cultures. Oocysts have a spectral signature, but it is orders of magnitude less bright. Normalizing the spectral signatures **(C)** confirms that uninfected and Sig M-negative COLO-680N cells have a similar profile, while oocysts and Sig M-positive COLO-680N cells have a similar profile. Using spectral unmixing, it is possible to create an autofluorescent tag for Sig M **(D)** that detects a signal from infected cultures by combining light across the full spectrum. This signature is absent from COLO-680N cells infected with *Salmonella*—one of two duplicate *Salmonella* cultures is shown **(E)**.

We postulated that measuring unstained *Cryptosporidium*-infected cultures on a spectral cytometer would allow us to create a normalized auto-fluorescent signature for Sig M on COLO-680N cells which we could compare with the normalized auto-fluorescent signature from uninfected COLO-680N cells, oocysts, and sporozoites. Using a three-laser Cytek Aurora with Cytek Assay Settings (CAS) for settings for fluorescent detectors, we measured infected COLO-680N cultures from both *C. hominis* and *C. parvum* infections. The median fluorescent intensity (MFI) in each detector for uninfected COLO-680N cultures was measured and then normalized against the detector producing the maximum measured MFI for these cells to give the normalized auto-fluorescent signature (**Figure 5B**). This process was repeated for Sig M-positive cells, which were identified on the spectral cytometer by selecting the cells that were distinctly brightly fluorescent in a comparable detector to that determined to be most optimal from data in **Figures 4A, B** (*i*.*e*., with excitation at a wavelength of 405 nm, with emission collected at wavelength 447nm), which is shown in **Figure 5A**. Sig M-negative cells were also analyzed in this way (but taking the dimly fluorescent in the optimal Sig M detector; **Figure 5A**). We found that the normalized auto-fluorescent signatures of uninfected COLO-680N cultures and Sig M-negative cells were identical. Meanwhile, Sig M-positive cells have a separate auto-fluorescent signature with a different profile which is consistently different regardless of the species of *Cryptosporidium* (**Figure 5C**). Furthermore, the normalized auto-fluorescent signature of oocysts is very similar to that of Sig M-positive COLO-680N cells, suggesting that Sig M on COLO-680N cells is connected to *Cryptosporidium* (**Figure 5C**).

By comparing the normalized auto-fluorescent signature, it is possible to compare the spectral similarities between biological particles, but this masks the absolute amounts of fluorescent light inherent to such particles. While oocysts and Sig M-positive cells have a similar auto-fluorescent signature, the median amounts of light that comprise this signature are vastly different, with oocysts having approximately 1/100th of the amount of signal of Sig M-positive COLO-680N cells (**Figure 5B**). This is to be expected as oocysts are a smaller particle and thus take less time to pass through the laser beam, thus generating less fluorescence than COLO-680N cells. Meanwhile, sporozoites are so small that it is not possible to reliably detect an auto-fluorescent signature that is distinguishable from fluorescence background derived from noise signals in the cytometer with these instrument settings (CAS) (data not shown).

We speculated that the spectral cytometer could be used to calculate an integrated Sig M across all the fluorescent detectors by using a Sig M-positive sample as a reference control to allow spectral unmixing in the cytometer software (Spectroflo™), sometimes described as generating an auto-fluorescent tag. **Figure 5D** demonstrates that Sig M can indeed be unmixed to create an auto-fluorescent tag (see also **Supplementary Figures S9**, **S10**). Additionally, *S. typhimurium*-infected cultures were examined for the presence of Sig M and, along with uninfected cultures, were found to be negative (**Figures 5D, E**; **Supplementary Figure S10**). Lastly, Sig M-positive cells from infected cultures were found to have elevated side scatter signals compared with uninfected cultures (**Supplementary Figure S11**), suggesting the increased internal complexity from these cells which could be due to the intramembranous location of the *Cryptosporidium* infection in these Sig M-positive cells.

Sig M can thus be measured as a normalized auto-fluorescent signature from infected COLO-680N cells (**Figures 5B, C**). This signature is the same regardless of the infectious agent (*C. parvum* or *C. hominis*) and closely matches the auto-fluorescent signature of unstained oocysts. Unmixing Sig M as an auto-fluorescent tag using spectral cytometry identifies a subset of cells in unstained infected cultures which is completely absent from uninfected cultures and *Salmonella*-infected cultures (**Figures 5D, E**). These results together describe a novel natural auto-fluorescent signature in cells from infected COLO-680N cultures that is related to *Cryptosporidium* as shown by the similarity of the normalized auto-fluorescent signatures.

### Gene expression time series

The relative expression of genes from our panel in samples infected with either *C. hominis* or *C. parvum* was compared with the respective sporozoites alone; untreated cells and *S.* Typhimurium-infected cells as controls were assessed using NanoString’s nCounter^®^ analysis system at 24, 48, 96, and 120 h post-inoculation.

**Figure 6:**
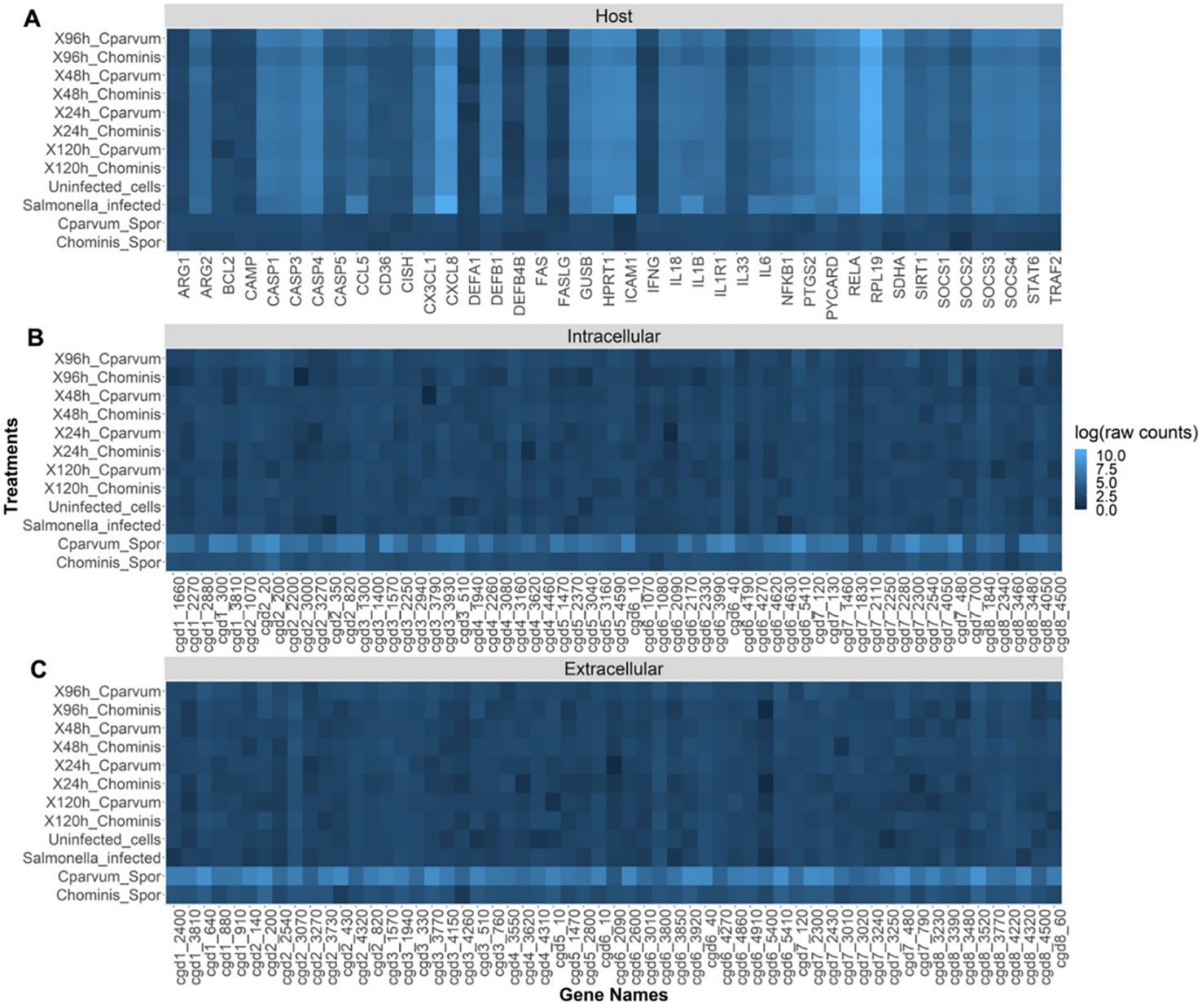
Heat map showing the relative abundance (log of raw counts) of each mRNA transcript (gene names) in each sample (treatments). **(A)** Expression of host (human) genes. **(B)** *Cryptosporidium* genes thought to be expressed at high levels intracellularly. **(C)** *Cryptosporidium* genes thought to be expressed at high levels extracellularly.

Compared with the host genes, the parasite genes were expressed at very low levels in infected cells (**Figure 6**). The mRNA counts of parasite genes in the infected cells and the controls (uninfected cells and *S.* Typhimurium-infected cells) were also similar. The raw mRNA counts (**Supplementary Table S5**) provide a clearer picture of this lack of variation. The sporozoites showed a relatively high expression of all the parasite genes compared with the infected cells (**Figure 6**). This is particularly notable because 48 of the parasite genes were selected based on their intracellular expression characteristics. The sporozoites also showed practically no significant expression of host genes, which verifies the specificity of the panel. From the results, there appeared to be a higher expression of parasite genes in the *C. parvum* sporozoites compared with the *C. hominis* sporozoites.

## Discussion

Most cell lines capable of culturing *Cryptosporidium* only allow limited progression through its life cycle and die after about 3 to 4 days post-inoculation (Karanis, 2018a). COLO-680N, an esophageal cell line, allows the long-term cultivation of the parasite without the need for specialized media or costly, complicated mechanical structures such as hollow fiber culture systems (Bones et al., 2019). COLO-680N cells are used here for the cultivation of both *C. hominis* and *C. parvum* in conjunction with Sporo-Glo™ and flow cytometry, showing that it is possible to identify and count the key life stages in this *in vitro* model for *Cryptosporidium* infection (**Figure 1**). With the substantial dynamic range of current flow cytometers, it is possible to identify *Cryptosporidium* sporozoites, shells, oocysts, and host cells in the same sample measurement. (King et al. 2009) show that flow cytometry can identify sporozoites, shells, and oocysts, and Sonzogni-Desautels et al. (2019) show that flow cytometry can detect oocysts from host cell material, while the data presented here take this further to detect all four entities simultaneously. Furthermore, both cytometers used in this study (Cytek^®^ Aurora and BD FACSVerse™) can determine the absolute particle count per milliliter during the measurement (shown for sporozoites and oocysts in **Supplementary Figure S1**), further conveying the insights possible in this model.

Sporo-Glo™ was generated against *C. parvum* and has been validated against the parasite (Boxell et al., 2008). It has also been used for the detection of other *Cryptosporidium*, including *C. hominis* (Hijjawi et al., 2010; Karanis, 2018b). In our assay, we saw a clear signal on sporozoites and oocysts from both *C. hominis* and *C. parvum* with no discernible difference in signal intensity (data not shown). Our first studies showed a population of cells from infected cultures producing a positive signal in the FITC detector (for Sporo-Glo™) on the traditional flow cytometer, the FACSVerse ™, leading us to initially assume that the signal detected was a result of the recognition of *Cryptosporidium* antigens in host cells by Sporo-Glo™. As further support for infection, cells from infected cultures showed IL-8 secretion by fluorescence microscopy (**Supplementary Figure S5**), which is a hallmark of an immune response to *Cryptosporidium* (Laurent, 1997). Nevertheless, the numbers of Sporo-Glo™-positive host cells from *C. hominis* infection were low, with less than 1/10 cells positive for this signal (**Figure 2**). The infected COLO-680N cultures were examined by flow cytometry at 48 h after infection. *C. hominis* and *C. parvum* may be at different points of their infectious cycle at this point, such that antigens recognized by Sporo-Glo™ are not readily available in host cells infected with *C. hominis*. Other differences between *C. hominis* and *C. parvum* may explain this result, but the most parsimonious reason is that the antibodies present in Sporo-Glo™ have a stronger binding affinity to epitopes in *C. parvum* antigens compared with *C. hominis* due to Sporo-Glo™ being generated against *C. parvum* sporozoites.

We observed a striking increase in autofluorescence in some cells from infected COLO-680N cultures. Typically, traditional flow cytometry measures fluorescence by using individual detectors for each fluorophore [such that the bulk of the fluorescence for a particular fluorochrome (*e*.*g*., FITC) will be measured in a single fluorescence detector], but despite this, if a particle has a dramatic increase in auto-fluorescence across a broad range of wavelengths of light, then one can expect to see an increase in the proportion of cells with this auto-fluorescence in a wide number of detectors. Indeed this is what we found. Naming this auto-fluorescent signal Sig M, we found a substantial proportion of Sig M-positive cells present in many detectors from infected cultures, even in the FITC detector (in the absence of Sporo-Glo™). It was therefore possible that the “FITC” signal detected from Sporo-Glo™-stained infected cultures was, in fact, derived from Sig M and was not a consequence of a specific interaction of Sporo-Glo™ with the host cells. Using a ratio of the signal of FITC light in the uninfected and infected cultures with and without Sporo-Glo™, we found that for *C. parvum* infection this ratio was noticeably higher in the presence of Sporo-Glo™. Sporo-Glo™ and Sig M are therefore contributing together to the FITC signal in the Sporo-Glo™ *C. parvum*-infected culture result. In contrast, upon examining *C. hominis*-infected cultures, the picture is far less clear, with only a small or non-existent increase in this ratio with the addition of Sporo-Glo™ seen in three separate experiments (**Figure 4**, **Supplementary Figures S7**, **S8**). Therefore, this could suggest that the signal detected in the FITC detector here following *C. hominis* infection is largely a consequence of Sig M, with little contribution from Sporo-Glo™-specific staining. Indeed it is interesting to note that proportions of Sig M-positive cells are generally higher in *C. hominis* infection as opposed to *C. parvum* infection. Given *C. hominis*’ adaptation to human cells, a higher level of infectivity might be expected from this species in such a comparison.

Sig M has only been investigated using flow cytometry, and it may be informative to explore how well fluorescent microscopy can identify such cells in the absence of Sporo-Glo™ staining to compare with data showing Sporo-Glo™-positive cells (**Figure 3**) by using fluorescent microscopy. We reasoned that the real power of measuring Sig M might come from being able to interrogate this intriguing new signal of parasite and host cells using a spectral cytometer. The unique feature of spectral cytometry is the ability to collect data across close to the entire rainbow of light. This is achieved by a large number of detectors, each of which has a fluorescence filter optimized to detect a discrete portion of the rainbow so that almost the whole rainbow is measured, while traditional flow cytometry does not tend to allow this. Sig M fluoresces across much of the violet and blue laser excited spectrum but is brightest in detectors from the violet laser at approximately 508–542 nm (**Figures 5B, C**). Using the unmixing workflow available on the Cytekbio Spectroflo software, we were able to unmix Sig M to generate a full-spectrum Sig M tag which was present in COLO-680N-infected cultures but absent from *Salmonella*-infected cultures and uninfected cultures (**Figures 5D, E**). Spectral cytometry is now available on cell sorting platforms (*e*.*g*., CytekBio Aurora CS), which should facilitate our ability to purify Sig M-positive cells and explore these cells further (*e*.*g*., by using fluorescent or transmission electron microscopy and targeted NanoString analysis) in the future. Because Sig M-positive COLO-680N cells increase in frequency with increasing MOI and because their normalized spectral signature is very similar to oocysts, we postulate that Sig M is a natural stain-free marker of infection in host cells. By exploring these cells further using spectral cytometry, it may be possible to confirm this by combining Sporo-Glo™ detection with Sig M. A careful experimental design to ensure that all stained and unstained samples receive the same buffer treatment to standardize changes to fluorescence caused by these steps will be necessary, and trialing anti-FITC antibodies conjugated to far red fluorophores to circumvent the impact of Sig M’s fluorescence in the “FITC” spectrum of Sporo-Glo™ should be beneficial. Oocysts that have attached to the exterior of host cells might also explain Sig M; however, there are extensive wash steps in the protocol to prepare the cells for analysis which may make this explanation less likely.

We do not know the source of Sig M. Prokaryotic and eukaryotic cells can both exhibit intrinsic natural fluorescence due to metabolites and cellular structural components. A previous work has shown that intrinsic cellular autofluorescence increases during stress in prokaryotes (*E. coli*) and in eukaryote (yeast and human) cell lines, suggesting that the processes are evolutionarily conserved (Surre et al, 2018). The switching between life stages and intra- and extracellular phases is likely stressful. Flavins and nicotinamide-adenine dinucleotide have been studied most because they are responsible for most cytoplasmic fluorescence and because they play key roles in cell metabolism, but the spectral profile here does not fit flavins or flavoproteins (Benson et al., 1979). More work is therefore required to determine the source. Given the spectral similarity between Sig M on some COLO-680N cells from infected cultures and oocysts, we can, however, speculate that Sig M might be a direct consequence of detecting parasitic products within COLO-680N cells. If sporozoites are also fluorescent, not detected here perhaps due to the overall settings on the spectral cytometer (CAS) being optimized for large mammalian cells such as lymphocytes, their abundance within the cytoplasmic membrane might explain the presence of Sig M from infected COLO-680N cultures.

Detecting oocysts in water samples is an important task for environmental monitoring. We previously reported that we rarely detect more than a few oocytes per 100 L of water with existing methods (Phiri et al, 2020). Given the speed and sensitivity of spectral cytometry, utilizing Sig M in a method to detect oocysts in water samples could provide a precise and sensitive method for the detection of environmental *Cryptosporidium* without the need for staining with fluorescent antibodies (Adeyemo et al., 2019), especially if some of the previous technical issues can be overcome (*e*.*g*., Lepesteur et al., 2003).

The variability which is present in the *Cryptosporidium* products available for establishing infection in our system may have played a role in some of the lower proportions of infected cells detected in some experiments. It became challenging to collect *C. hominis* samples following border closures enacted during the COVID-19 pandemic in New Zealand (Knox et al, 2021). Overall, hand hygiene and mask wearing practices may have impacted on the availability of *C. parvum* samples as well. This meant that the age of some of the stored *Cryptosporidium* samples was higher in some experiments. Being able to enumerate *Cryptosporidium* products present in the infectious ecosystem (**Supplementary Figure S1**) permitted us to observe that the numbers of these were much higher in the early optimization experiments of the model (**Supplementary Figure S1** and data not shown) conducted prior to the COVID-19 pandemic. We noted higher amounts of infection of host cells (**Figure 2** and data not shown) compared with experiments post-March 2020 where lower levels of infection in host cells were noted (**Figures 4**, **5** and **Supplementary Figures S2**-**S4**) along with reduced evidence of sporozoites in cultures (data not shown). Future work on this model could involve incorporating assays to validate *Cryptosporidium* viability prior to infection to control for this effect.

Using NanoString, the expression of these genes in the COLO-680N cell line was assessed, comparing it with sporozoites of both *C. parvum* and *C. hominis*. The raw counts showed very low levels of the expression of *Cryptosporidium*-specific genes in all the infected cell cultures, approaching the limit of detection of the nCounter^®^ machine. Similar to data from a similar study conducted by Lippuner et al. (2018), all the parasite genes were expressed at higher levels in sporozoites compared with the infected cells. Here cytometry and microscopy provide evidence of intracellular infection, but the very small population might mean that the increases in gene expression are diluted. The differences in the expression of parasite genes between *C. parvum* and *C. hominis* sporozoites could be due to the relatively low efficiency of excystation of the *C. hominis* oocysts (a common problem when using oocysts purified from human fecal samples) or the lower specificity of mRNA probes due to most studies on the transcriptome of *Cryptosporidium* having been conducted using *C. parvum*.

Lippuner et al. (2018) analyzed the transcriptome of *C. parvum* during infection using both *in vitro* and *in vivo* platforms. The results of the analysis of the data from that study showed significant differences in the most highly expressed genes between both culture platforms. This suggests that the host exerts significant pressure on which genes are expressed by the parasite. This is to be expected given that *Cryptosporidium* relies on the host for most of its metabolic processes (Cacció and Widmer, 2013). Therefore, *Cryptosporidium* cultured in different cell lines may show differing patterns of gene expression. COLO-680N is an esophageal cell line, while HCT-8 is a colorectal cell line, and while both cell lines are epithelial, the organ of origin might produce differences in gene expression. This limitation could be solved in future studies by increasing the infectious dose and amplification of the *Cryptosporidium* RNA to allow for an accurate analysis of the transcriptome of the parasite intracellularly and extracellularly. In addition, methods such as RNAseq, which does not limit the number of genes observed, cell sorting by flow cytometry to isolate target cells, or other laboratory approaches to isolate target cells may be used. Cell sorting by flow cytometry, however, comes with additional challenges on many systems, such as needing to fix the cells for biosafety purposes, which limits the use of some downstream assays that are sensitive to fixation. Further research should analyze the complete transcriptome of the parasite in the COLO-680N cell line across its life cycle using multiple *Cryptosporidium* species that have been found in humans.

In conclusion, this study presents the foundation of an *in vitro* assay potentially capable of assessing the infectivity of *C. parvum* and *C. hominis* in the COLO-680N cell line and suggests that it is possible to do so without the use of a fluorescent antibody. Following further validation and refinement, this system has the potential to serve as a platform for the testing of new molecules and drugs for the treatment of cryptosporidiosis and provide new insights into the disease mechanisms of this pathogen.

## Data availability statement

The data presented in the study are available in the CryptoDB database (http://cryptodb.org) and raw data and gene accession numbers given in the article **Supplementary Material**.

## Author contributions

DH conceived and designed the study with PO, JR, AH, NP, PB and JG-R. PO, JR, MK, NH and NV conducted the laboratory analyses. PO and JR performed the data analyses. PO wrote the original draft with JR and DH, and all co-authors contributed to editing. All authors contributed to the article and approved the submitted version.
