# Supplementary material for: A novel, stain-free, natural auto-fluorescent signal, Sig M, identified from cytometric and transcriptomic analysis of infectivity of Cryptosporidium hominis and Cryptosporidium parvum
Source: Front Cell Infect Microbiol. 2023 May 22;13:1178576. doi: 10.3389/fcimb.2023.1178576 (PMC10239843; doi:10.3389/fcimb.2023.1178576)
Supplement: Supplementary file 1 [file DataSheet_1.pdf]

Supplementary Material

A novel, stain-free, natural auto-fluorescent signal, Sig M, identified from cytometric and transcriptomic analysis of infectivity of *Cryptosporidium hominis* and *Cryptosporidium parvum*

Paul Ogbuigwe[1†], Joanna M Roberts[2†], Matthew A Knox[1], Axel Heiser[3], Anthony Pita[1], Neville A Haack[3], Juan Carlos Garcia-Ramirez[1], Niluka Velathanthiri[1], Patrick J Biggs[1], Nigel P French[1], David T S Hayman[1*]

*** Correspondence:** d.t.s.hayman@massey.ac.nz
† Equal contribution and first authorship

Supplementary Figure 1. Detection of counts/well of oocysts and sporozoites from cultures containing infected and uninfected COLO-680N cells following 48hr incubation with *Cryptosporidium*. Sporozoites and oocysts were identified (Fig 1) and counted using the volume measured during acquisition on the FACSVerse (equipped with thermic volume measurement).

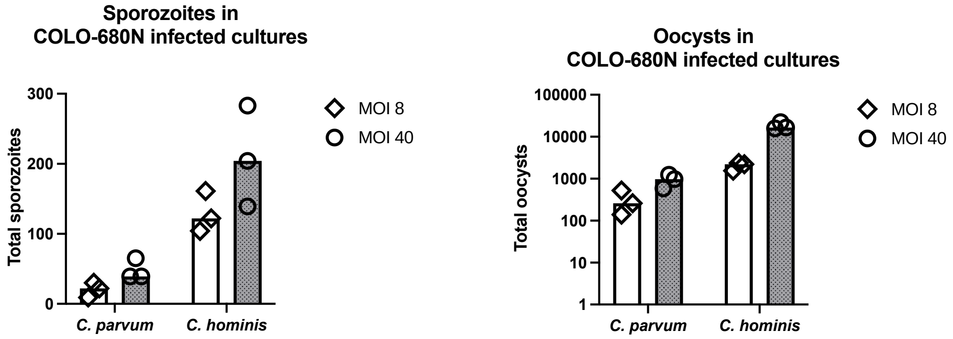

Supplementary Figure 2. Sporo-Glo™ positive COLO-680N cells are detectable in *C. parvum* infection and in increasing amounts with increasing MOI with *C. hominis*. (A) COLO-680N cells were infected at indicated MOI (dark blue dots) with either *C. parvum* (top two panels) or *C. hominis* (bottom two panels) and then fixed, permeabilized and stained with Sporo-Glo™. Uninfected cells stained with Sporo-Glo™ (light grey dots) are overlaid on each panel. Region defining Sporo-Glo™ positive cells is shown and percentage of cells in this region displayed. (B) Duplicates from (A) plotted as percent Sporo-Glo™ positive cells.

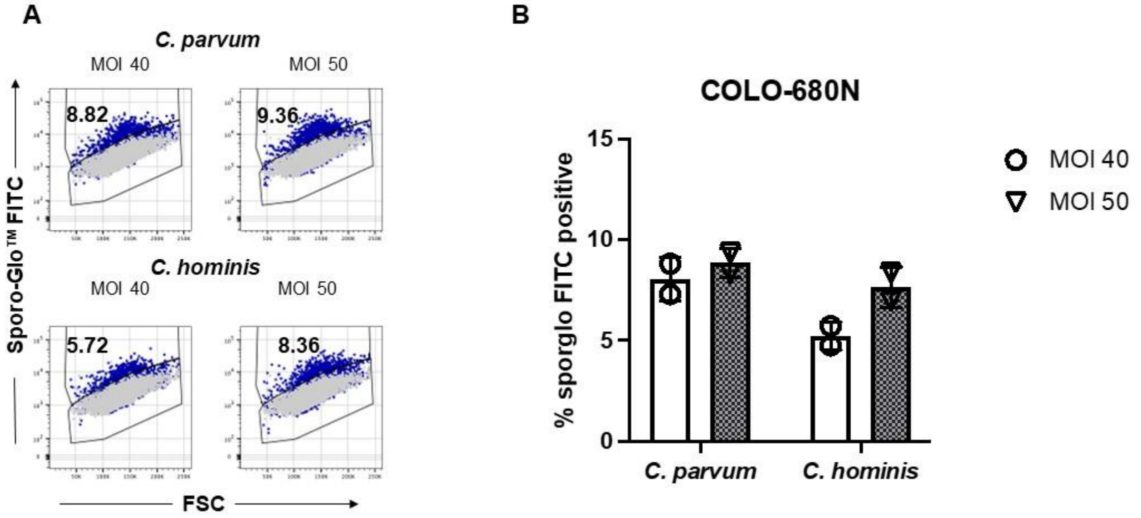

Supplementary Figure 3. Sporo-Glo™ positive COLO-680N cells are detectable at low levels regardless of MOI in *C. parvum* and *C. hominis* infection. (A) COLO-680N cells were infected at indicated MOI (dark blue dots) with either *C. parvum* (top three panels) or *C. hominis* (bottom three panels) and then fixed, permeabilized and stained with Sporo-Glo™ . Uninfected cells stained with Sporo-Glo™ (light grey dots) are overlaid on each panel, and shown separately to the right. Regions defining Sporo-Glo™ positive and negatives cells are shown and percentage of cells in each region displayed. (B) Single data points from (A) plotted as percent Sporo-Glo™ FITC positive cells.

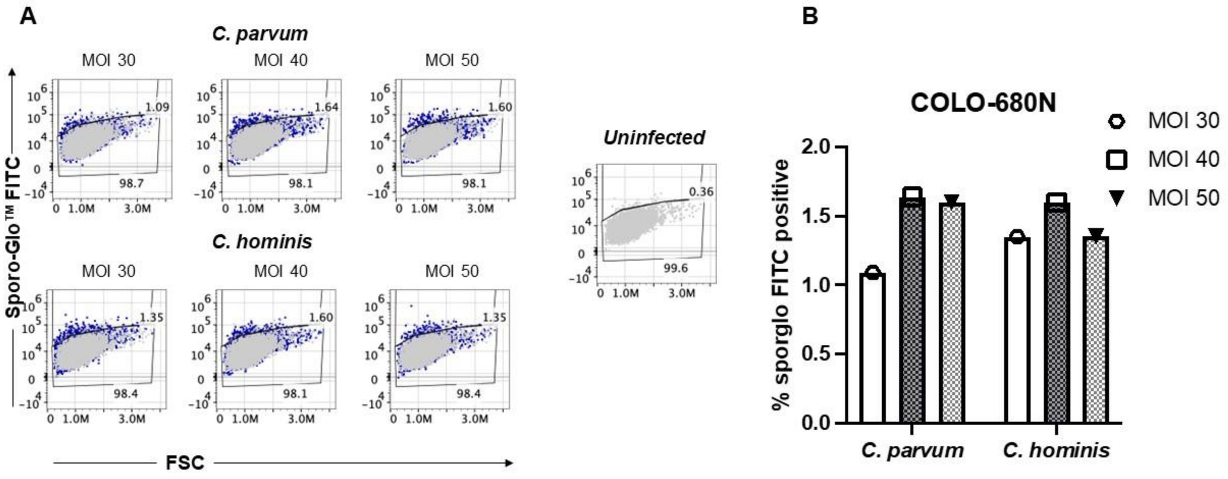

Supplementary Figure 4. Sporo-Glo™ positive COLO-680N cells are detectable at low levels with a trend to increasing proportions of positive cells with increasing MOI for *C. parvum* and *C hominis.* (A) COLO-680N cells were infected at indicated MOI (dark blue dots) with either *C. parvum* (top three panels) or *C. hominis* (bottom three panels) and then fixed, permeabilized and stained with Sporo-Glo™. Uninfected cells stained with Sporo-Glo™ (light grey dots) are overlaid on each panel and shown separately to the right. Regions defining Sporo-Glo™ positive and negatives cells are shown and percentage of cells in each region displayed. (B) Duplicates from (A) plotted as percent Sporo-Glo™ positive cells.

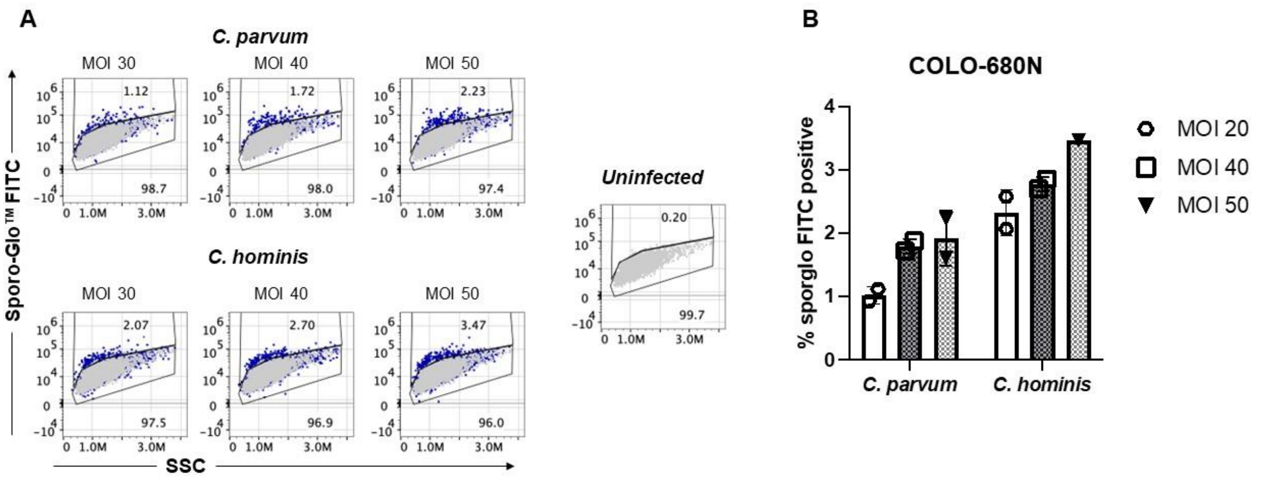

Supplementary Figure 5. Confocal microscopy image of *C. parvum*-infected COLO-680N cells expressing IL-8. Uninfected cells are displayed in A, infected cells are displayed in B. The nucleus is stained blue with DAPI, and the red/orange dots represent IL-8 molecules tagged with a fluorescent antibody. 72 days old *C. parvum* IIaA18G2R1 excysted oocysts were used for infection in this experiment. Addition of Brefeldin A 12 hrs before fixing resulted in some cell death and shape distortion in some cells. Scale bar represents 14 µm.

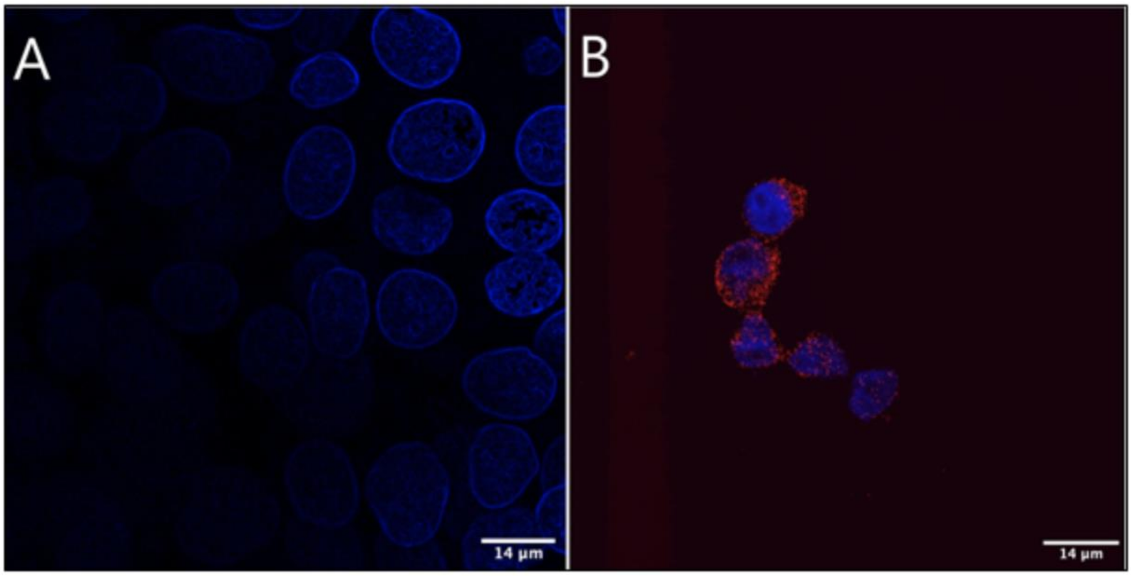

Supplementary Figure 6. Sig M-style fluorescence detected in an early experiment where all infected cultures were fixed, permeabilized and fully stained (no infected unstained cells to allow discreet measurement of Sig M). Infected, stained COLO-680N cultures (Sporo-Glo™, FVDefluor780) contain a population of SigM positive cells identifiable with a range of emission and excitation optics. Note, presence of Sporo-Glo™ will likely influence signal detected in some spectrally proximal detectors (e.g., lambda568nm).

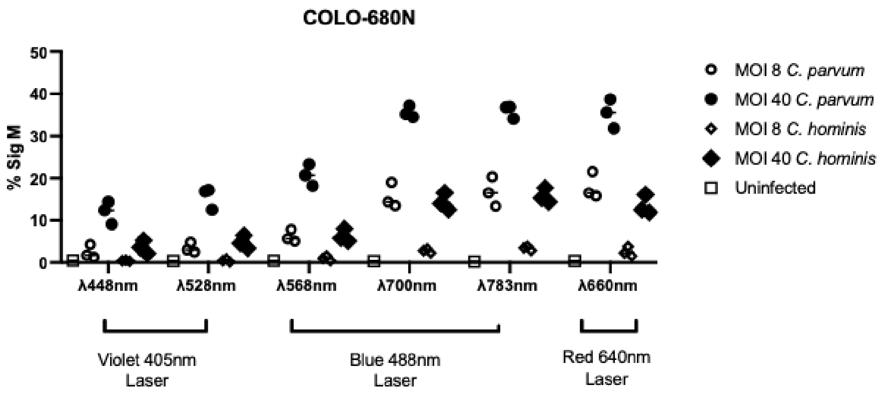

Supplementary Figure 7. Ratio of FITC/lambda 528nm light for Sig M high cells vs Sig M low cells may demonstrate a small increase in favour of a specific Sporo-Glo™ signal for parvum infected cultures while *C. hominis* infected cultures may show a similar ratio regardless of Sporo-Glo™ staining. Each circle represents a ratio calculated from an individual infected COLO-680N culture.

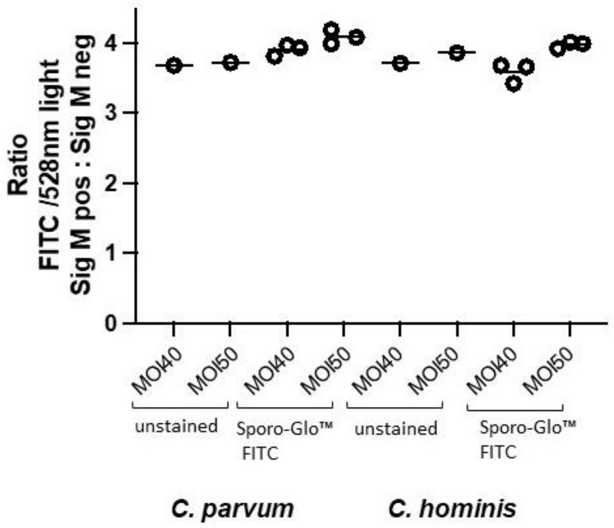

Supplementary Figure 8. Ratio of FITC/lambda 528nm light for Sig M high cells vs Sig M low cells demonstrates a small increase in favour of a specific Sporo-Glo™ signal for *C. parvum* infected cultures while *C. hominis* infected cultures may show a similar ratio regardless of Sporo-Glo™ staining. Each circle represents a ratio calculated from an individual infected COLO-680N culture.

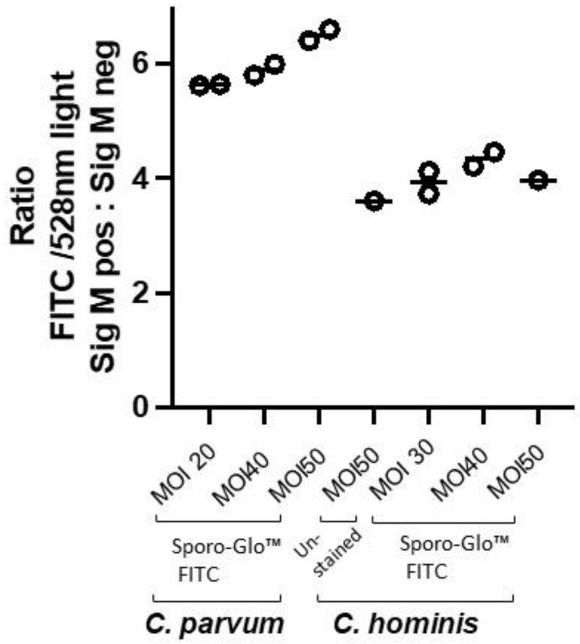

Supplementary Figure 9. Sig M is detectable in *C. hominis* infected cultures, showing a trend of increasing frequency of Sig M positive cells with increasing MOI.

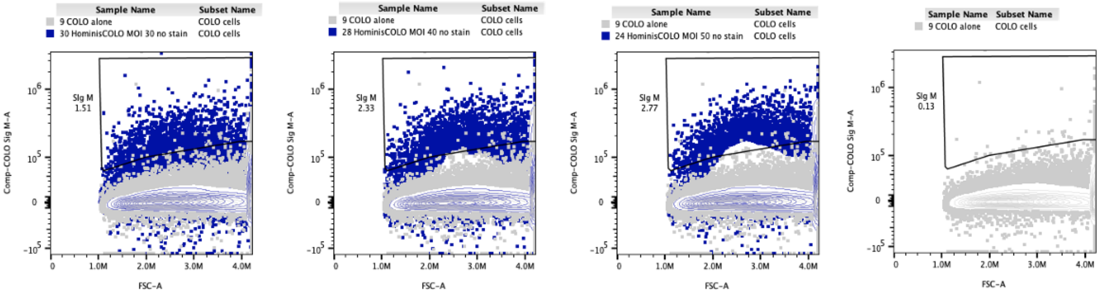

Supplementary Figure 10. Sig M is detectable in *C. hominis* and *C. parvum* infected cultures, but not in *Salmonella* infected cultures (one representative of duplicate *Salmonella* infection shown)

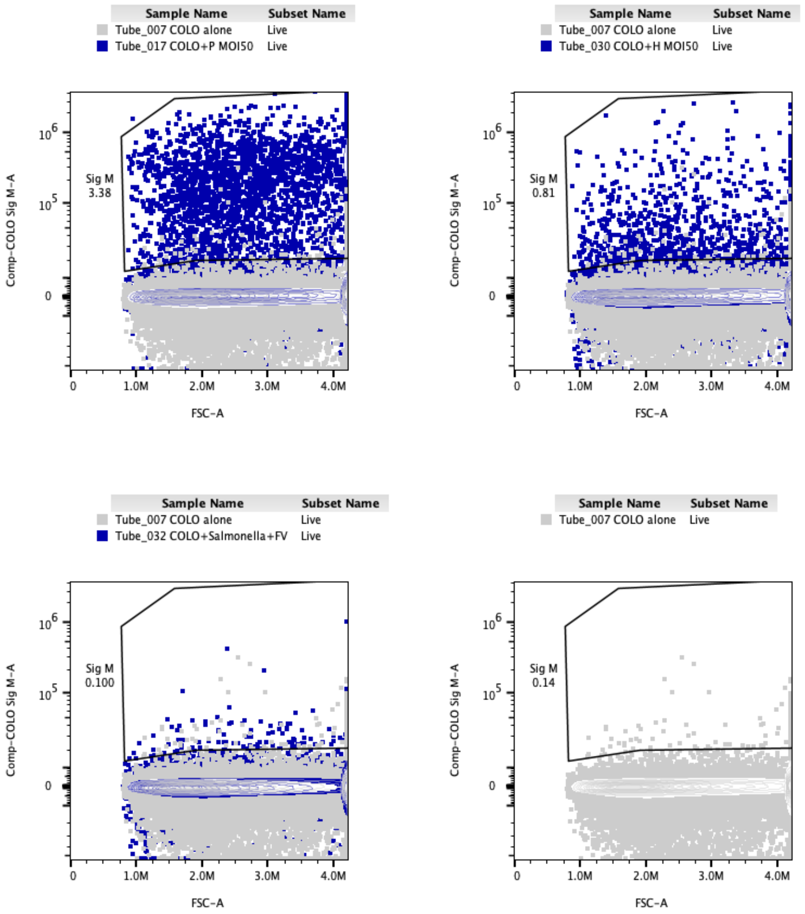

Supplementary Figure 11. Side scatter of Sig M positive cells is too bright to be clearly resolved using these instrument settings (CAS), showing that Sig M positive COLO-680N cells are likely more granular than the uninfected COLO-680N cells shown for comparison (from uninfected cultures) (representative of two separate experiments and both species of *Cryptosporidium*)

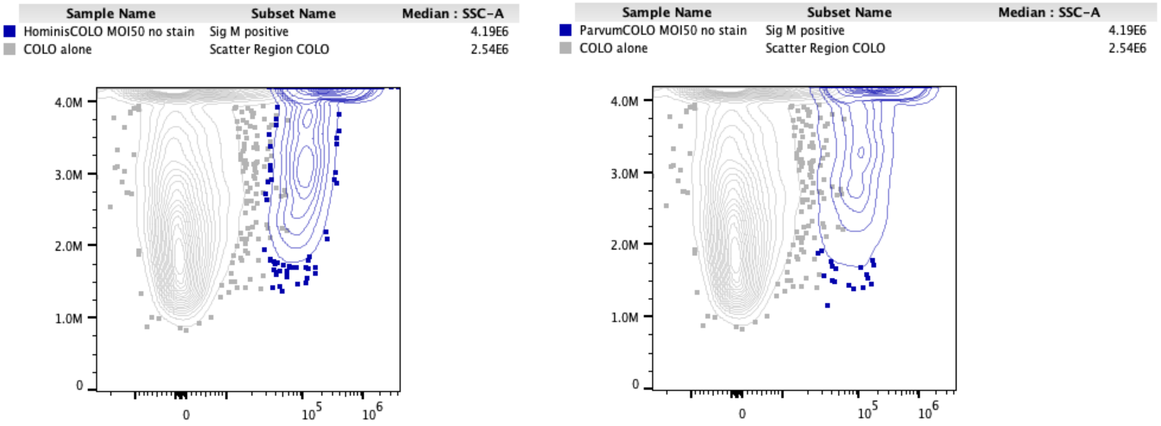

Supplementary Table 1. List of target genes used in this study.

Gene ID	Accession	Flags	Species
**cgd2_3270**	XM_626517.1	Housekeeping Gene	*Cryptosporidium parvum*
**cgd5_3160**	XM_001388245.1	Housekeeping Gene	*Cryptosporidium parvum*
**cgd6_2090**	XM_627569.1	Housekeeping Gene	*Cryptosporidium parvum*
**cgd6_4270**	XM_627759.1	Housekeeping Gene	*Cryptosporidium parvum*
**cgd_2_140**	XM_625383.1		*Cryptosporidium parvum*
**cgd1_1660**	XM_628006.1		*Cryptosporidium parvum*
**cgd1_2270**	XM_628059.1		*Cryptosporidium parvum*
**cgd1_2400**	XM_628069.1		*Cryptosporidium parvum*
**cgd1_2880**	XM_628107.1		*Cryptosporidium parvum*
**cgd1_300**	XM_627890.1		*Cryptosporidium parvum*
**cgd1_3810**	XM_628192.1		*Cryptosporidium parvum*
**cgd1_640**	XM_627916.1		*Cryptosporidium parvum*
**cgd1_880**	XM_001388111.1		*Cryptosporidium parvum*
**cgd1_910**	XM_627937.1		*Cryptosporidium parvum*
**cgd2_1070**	XM_626335.1		*Cryptosporidium parvum*
**cgd2_20**	XM_625373.1		*Cryptosporidium parvum*
**cgd2_200**	XM_625389.1		*Cryptosporidium parvum*
**cgd2_2200**	XM_626425.1		*Cryptosporidium parvum*
**cgd2_2540**	XM_626456.1		*Cryptosporidium parvum*
**cgd2_3000**	XM_626494.1		*Cryptosporidium parvum*
**cgd2_3070**	XM_626501.1		*Cryptosporidium parvum*
**cgd2_350**	XM_625403.1		*Cryptosporidium parvum*
**cgd2_3730**	XM_626558.1		*Cryptosporidium parvum*
**cgd2_430**	XM_625410.1		*Cryptosporidium parvum*
**cgd2_4320**	XM_626611.1		*Cryptosporidium parvum*
**cgd2_820**	XM_626315.1		*Cryptosporidium parvum*
**cgd3_1300**	XM_626705.1		*Cryptosporidium parvum*
**cgd3_1400**	XM_626715.1		*Cryptosporidium parvum*
**cgd3_1570**	XM_001388065.1		*Cryptosporidium parvum*
**cgd3_1940**	XM_626765.1		*Cryptosporidium parvum*
**cgd3_2250**	XM_626792.1		*Cryptosporidium parvum*
**cgd3_2940**	XM_626851.1		*Cryptosporidium parvum*
**cgd3_330**	XM_001388055.1		*Cryptosporidium parvum*
**cgd3_3770**	XM_626924.1		*Cryptosporidium parvum*
**cgd3_3790**	XM_626926.1		*Cryptosporidium parvum*
**cgd3_3930**	XM_626938.1		*Cryptosporidium parvum*
**cgd3_4150**	XM_001388094.1		*Cryptosporidium parvum*

**cgd3_4260**	XM_626969.1		*Cryptosporidium parvum*
**cgd3_510**	XM_626636.1		*Cryptosporidium parvum*
**cgd3_760**	XM_626659.1		*Cryptosporidium parvum*
**cgd4_1940**	XM_625788.1		*Cryptosporidium parvum*
**cgd4_2260**	XM_625816.1		*Cryptosporidium parvum*
**cgd4_3080**	XM_625892.1		*Cryptosporidium parvum*
**cgd4_3160**	XM_625900.1		*Cryptosporidium parvum*
**cgd4_3550**	XM_625931.1		*Cryptosporidium parvum*
**cgd4_3620**	XM_625938.1		*Cryptosporidium parvum*
**cgd4_4310**	XM_625446.1		*Cryptosporidium parvum*
**cgd4_4460**	XM_625460.1		*Cryptosporidium parvum*
**cgd5_10**	XM_625966.1		*Cryptosporidium parvum*
**cgd5_1470**	XM_626094.1		*Cryptosporidium parvum*
**cgd5_2370**	XM_626174.1		*Cryptosporidium parvum*
**cgd5_2800**	XM_626216.1		*Cryptosporidium parvum*
**cgd5_3040**	XM_626236.1		*Cryptosporidium parvum*
**cgd5_4590**	XM_625307.1		*Cryptosporidium parvum*
**cgd6_10**	XM_625341.1		*Cryptosporidium parvum*
**cgd6_1070**	XM_627479.1		*Cryptosporidium parvum*
**cgd6_1080**	XM_627480.1		*Cryptosporidium parvum*
**cgd6_2170**	XM_627575.1		*Cryptosporidium parvum*
**cgd6_2330**	XM_627590.1		*Cryptosporidium parvum*
**cgd6_2600**	XM_627613.1		*Cryptosporidium parvum*
**cgd6_3010**	XM_001388297.1		*Cryptosporidium parvum*
**cgd6_3800**	XM_627719.1		*Cryptosporidium parvum*
**cgd6_3850**	XM_627723.1		*Cryptosporidium parvum*
**cgd6_3920**	XM_627729.1		*Cryptosporidium parvum*
**cgd6_3990**	XM_001388307.1		*Cryptosporidium parvum*
**cgd6_40**	XM_625344.1		*Cryptosporidium parvum*
**cgd6_4190**	XM_627752.1		*Cryptosporidium parvum*
**cgd6_4620**	XM_627791.1		*Cryptosporidium parvum*
**cgd6_4630**	XM_627792.1		*Cryptosporidium parvum*
**cgd6_4860**	XM_627813.1		*Cryptosporidium parvum*
**cgd6_4910**	XM_627816.1		*Cryptosporidium parvum*
**cgd6_5400**	XM_627858.1		*Cryptosporidium parvum*
**cgd6_5410**	XM_627859.1		*Cryptosporidium parvum*
**cgd7_120**	XM_001388325.1		*Cryptosporidium parvum*
**cgd7_130**	XM_628206.1		*Cryptosporidium parvum*
**cgd7_1460**	XM_628328.1		*Cryptosporidium parvum*
**cgd7_1830**	XM_628358.1		*Cryptosporidium parvum*
**cgd7_2110**	XM_628385.1		*Cryptosporidium parvum*
**cgd7_2250**	XM_628397.1		*Cryptosporidium parvum*

**cgd7_2280**	XM_628400.1		*Cryptosporidium parvum*
**cgd7_2300**	XM_628401.1		*Cryptosporidium parvum*
**cgd7_2430**	XM_628414.1		*Cryptosporidium parvum*
**cgd7_2540**	XM_628424.1		*Cryptosporidium parvum*
**cgd7_3010**	XM_628466.1		*Cryptosporidium parvum*
**cgd7_3020**	XM_628467.1		*Cryptosporidium parvum*
**cgd7_3240**	XM_001388357.1		*Cryptosporidium parvum*
**cgd7_3250**	XM_628487.1		*Cryptosporidium parvum*
**cgd7_4050**	XM_628560.1		*Cryptosporidium parvum*
**cgd7_480**	XM_628236.1		*Cryptosporidium parvum*
**cgd7_700**	XM_001388332.1		*Cryptosporidium parvum*
**cgd7_790**	XM_628266.1		*Cryptosporidium parvum*
**cgd8_1840**	XM_001388389.1		*Cryptosporidium parvum*
**cgd8_2340**	XM_627137.1		*Cryptosporidium parvum*
**cgd8_3230**	XM_627219.1		*Cryptosporidium parvum*
**cgd8_3390**	XM_627234.1		*Cryptosporidium parvum*
**cgd8_3460**	XM_627239.1		*Cryptosporidium parvum*
**cgd8_3480**	XM_627241.1		*Cryptosporidium parvum*
**cgd8_3520**	XM_627245.1		*Cryptosporidium parvum*
**cgd8_3770**	XM_627269.1		*Cryptosporidium parvum*
**cgd8_4050**	XM_627296.1		*Cryptosporidium parvum*
**cgd8_4220**	XM_627312.1		*Cryptosporidium parvum*
**cgd8_4320**	XM_627320.1		*Cryptosporidium parvum*
**cgd8_4500**	XM_627336.1		*Cryptosporidium parvum*
**cgd8_60**	XM_625470.1		*Cryptosporidium parvum*
**ARG1**	NM_000045.3		*Homo sapiens*
**ARG2**	NM_001172.3		*Homo sapiens*
**BCL2**	NM_000657.2		*Homo sapiens*
**CAMP**	NM_004345.3		*Homo sapiens*
**CASP1**	NM_001223.3		*Homo sapiens*
**CASP3**	NM_004346.3		*Homo sapiens*
**CASP4**	NM_001225.3		*Homo sapiens*
**CASP5**	NM_004347.1		*Homo sapiens*
**CCL5**	NM_002985.2		*Homo sapiens*
**CD36**	NM_000072.3		*Homo sapiens*
**CISH**	NM_145071.2		*Homo sapiens*
**CX3CL1**	NM_002996.3		*Homo sapiens*
**CXCL8**	NM_000584.2		*Homo sapiens*
**DEFA1**	NM_004084.2		*Homo sapiens*
**DEFB1**	NM_005218.3		*Homo sapiens*
**DEFB4B**	NM_001205266.1		*Homo sapiens*
**FAS**	NM_000043.4		*Homo sapiens*
**FASLG**	NM_000639.1		*Homo sapiens*

**GUSB**	NM_000181.3		*Homo sapiens*
**HPRT1**	NM_000194.1		*Homo sapiens*
**ICAM1**	NM_000201.2		*Homo sapiens*
**IFNG**	NM_000619.2		*Homo sapiens*
**IL18**	NM_001562.3		*Homo sapiens*
**IL1B**	NM_000576.2		*Homo sapiens*
**IL1R1**	NM_001320984.1		*Homo sapiens*
**IL33**	NM_033439.2		*Homo sapiens*
**IL6**	NM_000600.3		*Homo sapiens*
**NFKB1**	NM_003998.2		*Homo sapiens*
**PTGS2**	NM_000963.1		*Homo sapiens*
**PYCARD**	NM_013258.3		*Homo sapiens*
**RELA**	NM_021975.2		*Homo sapiens*
**RPL19**	NM_000981.3		*Homo sapiens*
**SDHA**	NM_004168.1		*Homo sapiens*
**SIRT1**	NM_012238.4		*Homo sapiens*
**SOCS1**	NM_003745.1		*Homo sapiens*
**SOCS2**	NM_003877.3		*Homo sapiens*
**SOCS3**	NM_003955.3		*Homo sapiens*
**SOCS4**	NM_199421.1		*Homo sapiens*
**STAT6**	NM_003153.3		*Homo sapiens*
**TRAF2**	NM_021138.3		*Homo sapiens*

Supplementary Table 2. Fifty genes most highly expressed in oocysts/sporozoites from Matos et al (2019). (*) represents the number of orthologues within eupathDB genomes. (**) represents the number of OrthoMCL orthologous sequences.

Gene ID	Product Description	Ortholog Group	Ortholog count EupathDB*	Ortholog count total**
**cgd2_140**	Stress-associated_endoplasmic_reticulum_protein	OG5_130196	5	58
**cgd1_2400**	Uncharacterized_secreted_protein	OG5_222471	8	2
**cgd1_3810**	Uncharacterized_protein	OG5_194982	9	3
**cgd1_640**	Signal_peptide_region_containing_protein	OG5_194890	9	3
**cgd1_880**	Eukaryotic_initiation_factor_4A	OG5_126984	15	154
**cgd1_910**	AN1-type_and_A20-type_Zinc_finger	OG5_127624	13	112
**cgd2_200**	Uncharacterized_protein	OG5_130876	12	50
**cgd2_2540**	Glutaredoxin	OG5_126864	17	172
**cgd2_3070**	High_mobility_group_box_domain_containing_protein	OG5_126740	39	202
**cgd2_3730**	Glutathione_S-transferase_C-terminal_domain_containing_protein	OG5_180098	9	4
**cgd2_430**	Signal_peptide_containing_protein	OG5_222485	6	2
**cgd2_4320**	Thioredoxin/glutathione_reductase_selenoprotein	OG5_126785	23	189
**cgd2_820**	Translation_initiation_factor_SUI1	OG5_127384	12	124
**cgd3_1570**	Profilin	OG5_141746	11	14
**cgd3_1940**	Translationally_controlled_tumour_protein_associated_protein	OG5_127617	11	112
**cgd3_330**	unspecified product	OG5_129031	11	77
**cgd3_3770**	_Hsp90	OG5_126623	17	314
**cgd3_4150**	RNA_recognition_motif_domain_containing_protein	OG5_129225	10	73
**cgd3_510**	GDP-fucose_transporter	OG5_188054	8	3
**cgd3_760**	Ribosomal_protein_L7Ae/L30e/S12e/Gadd45	OG5_128156	12	97
**cgd4_3550**	Secreted_Kazal_domain-containing_protein	OG5_129460	24	69
**cgd4_3620**	Immunodominant_antigen_23393226	OG5_222616	7	2
**cgd4_4310**	Ring_finger_domain_containing_protein	OG5_191746	7	3
**cgd5_10**	Uncharacterized_Secreted_Protein	OG5_222658	7	2
**cgd5_1470**	Nucleoside_diphosphate_kinase	OG5_126708	22	214
**cgd5_2800**	Actin_depolymerizing_factor	OG5_127118	12	143
**cgd6_10**	Uncharacterized_protein	OG5_222668	7	2
**cgd6_2600**	Uncharacterized_protein	OG5_195457	9	3
**cgd6_3010**	Polyadenylate-binding_protein	OG5_126795	15	187
**cgd6_3850**	50S_ribosomal_protein_L30e-like	OG5_127322	15	127
**cgd6_3920**	Uncharacterized_protein_with_Tetratricopeptide-like_helical	OG5_175069	9	4
**cgd6_40**	Uncharacterized_protein	OG5_164335	7	6
**cgd6_4860**	DEAD/DEAH_box_helicase_with_GUCT_domain	OG5_128505	15	89
**cgd6_4910**	Zinc_finger_C3H1-type_domain_containing_protein	OG5_190023	11	3

**cgd6_5400**	Uncharacterized_Secreted_Protein	OG5_214933	6	2
**cgd6_5410**	Uncharacterized_Secreted_Protein	OG5_222709	7	2
**cgd7_2300**	Translation_elongation_factor_IF5A	OG5_126922	13	162
**cgd7_2430**	Translation_initiation_factor_eIF-5_Tif5p/ZnR+W2_domain-containing_protein	OG5_127779	13	106
**cgd7_3010**	Uncharacterized_protein	OG5_195622	9	3
**cgd7_3240**	RNA_polymerase_archaeal_subunit_P/eukaryotic_subunit_RPABC4	OG5_130124	7	59
**cgd7_3250**	Uncharacterized_Protein	OG5_195630	9	3
**cgd7_480**	L-lactate/malate_dehydrogenase	OG5_126911	29	164
**cgd7_790**	Uncharacterized_protein	OG5_169153	6	5
**cgd8_3230**	AP2/ERF_domain_containing_protein	OG5_195785	7	3
**cgd8_3480**	60S_ribosomal_protein_L34	OG5_127025	12	151
**cgd8_3520**	Uncharacterized_Secreted_Protein	OG5_135537	9	23
**cgd8_3770**	DNAJ_like_chaperone	OG5_126883	17	168
**cgd8_4220**	High_mobility_group_box_domain_containing_protein	OG5_144330	10	12
**cgd8_4320**	Uncharacterized_Protein	OG5_195816	9	3
**cgd8_60**	Uncharacterized_Protein	OG5_222757	7	2

Supplementary Table 3. Fifty genes most highly expressed in trophozoites/meronts from Matos et al (2019). (*) represents the number of orthologues within eupathDB genomes. (**) represents the number of OrthoMCL orthologous sequences.

Gene ID	Product Description	Ortholog Group	Ortholog count euk pathogens*	Ortholog count total**
**cgd1_1660**	60S_ribosomal_protein_L36	OG5_127134	12	141
**cgd1_2270**	40S_ribosomal_protein_S26	OG5_127138	12	141
**cgd1_2880**	Uncharacterized_protein	OG5_cpar|cgd1_2880	3	
**cgd1_300**	40S_ribosomal_protein_S21	OG5_127484	11	118
**cgd1_3810**	Uncharacterized_protein	OG5_194982	9	3
**cgd2_1070**	40S_ribosomal_protein_S25	OG5_126998	12	153
**cgd2_200**	Uncharacterized_protein	OG5_130876	12	50
**cgd2_2200**	Ribosomal_protein_L37	OG5_126918	12	163
**cgd2_3000**	40S_ribosomal_protein_S16	OG5_126930	12	161
**cgd2_350**	60S_ribosomal_protein_L39	OG5_127376	6	125
**cgd2_820**	Translation_initiation_factor_SUI1	OG5_127384	12	124
**cgd3_1300**	_60S_ribosomal_protein_L12	OG5_127022	12	151
**cgd3_1570**	Profilin	OG5_141746	11	14
**cgd3_2250**	60S_ribosomal_protein_L37A	OG5_127166	11	139
**cgd3_3790**	60S_ribosomal_protein_L19	OG5_126940	11	160
**cgd3_3930**	60S_ribosomal_protein_L27A	OG5_127016	11	152
**cgd3_510**	GDP-fucose_transporter	OG5_188054	8	3
**cgd4_2260**	_60S_acidic_ribosomal_protein_P0	OG5_127051	12	149
**cgd4_3080**	Ribosomal_protein_S27a_with_Zinc-binding_domain/ubiquitin/Zinc-binding_domain	OG5_127221	14	135
**cgd4_3160**	40S_ribosomal_protein_S3a	OG5_126852	12	175
**cgd4_3620**	Immunodominant_antigen_23393226	OG5_222616	7	2
**cgd5_1470**	Nucleoside_diphosphate_kinase	OG5_126708	22	214
**cgd5_2370**	60S_acidic_ribosomal_protein_L12/LP1-like_protein	OG5_126872	13	170
**cgd5_3040**	40S_ribosomal_protein_S7	OG5_127368	12	125
**cgd5_3160**	Actin	OG5_126595	23	421
**cgd6_10**	Uncharacterized_protein	OG5_222668	7	2
**cgd6_1070**	Uncharacterized_protein	OG5_128239	12	95
**cgd6_1080**	Glycoprotein_GP40	OG5_222676	7	2
**cgd6_2170**	60S_ribosomal_protein_L5	OG5_126688	12	222
**cgd6_2330**	Uncharacterized_protein	OG5_195453	9	3
**cgd6_3990**	Elongation_factor_1-alpha	OG5_126631	17	277
**cgd6_40**	Uncharacterized_protein	OG5_164335	7	6
**cgd6_4190**	60S_ribosomal_protein_L10/L16,_alpha/beta_hammerhead	OG5_126951	12	158
**cgd6_4620**	60S_ribosomal_protein_L26	OG5_126931	12	161

**cgd6_4630**	40S_ribosomal_protein_S8	OG5_127039	12	150
**cgd6_5410**	Uncharacterized_Secreted_Protein	OG5_222709	7	2
**cgd7_130**	Ribosomal_protein_S11	OG5_126681	12	225
**cgd7_1460**	40S_ribosomal_protein_S27	OG5_127008	11	153
**cgd7_1873**	Zinc-binding_ribosomal_protein_L44	OG5_126988	12	154
**cgd7_2110**	60S_ribosomal_proteins_L8/L2	OG5_126641	14	264
**cgd7_2250**	40S_ribosomal_protein_S3_KH2	OG5_126820	13	181
**cgd7_2280**	Ribosomal_protein_L40e	OG5_128257	27	95
**cgd7_2300**	Translation_elongation_factor_IF5A	OG5_126922	13	162
**cgd7_2540**	60S_ribosomal_protein_L35A	OG5_127072	12	147
**cgd7_4050**	Ribosomal_protein_L38	OG5_127448	11	120
**cgd7_480**	L-lactate/malate_dehydrogenase	OG5_126911	29	164
**cgd8_2340**	Cold-shock_DNA-binding_domain-containing__protein	OG5_126866	16	171
**cgd8_3480**	60S_ribosomal_protein_L34	OG5_127025	12	151
**cgd8_4050**	Ribosomal_protein_S29	OG5_127205	7	136
**cdg8_1840**	Ribosomal_protein_S4/S9	OG5_126983	13	154

Supplementary Table 4. Expression values of top 50 genes expressed in all *Cryptosporidium parvum* samples (including sporozoite data). The log2 values of the normalised read counts and the normalised read counts per kb (divided by transcript length) are listed for all libraries. Taken from Lippuner et al (2018).

**CryptoDB ID**	**Reads sporozoites (log2(x+1))**	**CryptoDB ID**	**Reads in vivo 2 days p.i. (log2(x+1))**	**CryptoDB ID**	**Reads in vivo 4 days p.i. (log2(x+1))**	**CryptoDB ID**	**Reads in vitro 2 days p.i. average (log2(x+1))**	**CryptoDB ID**	**Reads in vitro 4 days p.i. average (log2(x+1))**
cgd8_3520	15.96	cgd6_1080	14.15	cgd7_4020	14.16	cgd6_1080	14.39	cgd8_3520	13.92
cgd7_480	15.80	cgd6_2090	13.01	cgd6_1080	13.81	cgd8_3520	13.51	cgd7_480	13.90
cgd1_640	15.76	cgd7_4020	12.85	cgd8_3520	13.71	cgd7_480	13.40	cgd1_640	13.59
cgd6_5410	14.66	cgd8_3520	12.84	cgd6_2090	13.63	cgd1_640	13.17	cgd6_1080	13.42
cgd3_3770	14.34	cgd6_200	12.57	cgd6_200	13.59	cgd6_5410	12.60	cgd6_5410	12.88
cgd4_3550	14.20	cgd6_3990	12.23	cgd1_640	13.14	cgd3_510	12.20	cgd3_510	12.36
cgd8_3230	14.04	cgd3_3370	12.15	cgd3_3370	13.10	cgd4_3550	12.06	cgd4_3550	12.34
cgd1_3810	13.83	cgd6_5410	12.00	cgd6_5410	12.77	cgd3_3770	11.99	cgd3_3770	12.25
cgd3_510	13.80	cgd5_3160	11.94	cgd7_480	12.68	cgd6_200	11.98	cgd1_3810	12.07
cgd6_2600	13.69	cgd6_10	11.89	cgd5_3160	12.57	cgd1_3810	11.91	cgd8_3230	12.02
cgd2_200	13.67	cgd1_3810	11.79	cgd6_3990	12.28	cgd8_3230	11.68	cgd6_5400	11.91
cgd6_3920	13.65	cgd7_4810	11.75	cgd3_510	12.11	cgd6_5400	11.59	cgd6_10	11.64
cgd6_5400	13.65	cgd7_480	11.71	cgd5_1470	12.06	cgd6_2450	11.53	cgd2_3110	11.59
cgd6_3010	13.63	cgd4_3090	11.69	cgd4_3550	11.93	cgd2_3110	11.39	cgd6_3010	11.57
cgd7_2430	13.42	cgd8_4830	11.68	cgd1_3810	11.93	cgd6_3010	11.38	cgd6_3920	11.55

cgd2_20	13.38	cgd6_710	11.66	cgd2_3110	11.75	cgd6_3460	11.36	cgd6_2600	11.49
cgd2_3110	13.33	cgd5_1470	11.65	cgd6_4460	11.73	cgd6_3990	11.32	cgd8_1770	11.41
cgd6_10	13.31	cgd2_790	11.60	cgd3_1570	11.72	cgd6_10	11.25	cgd6_2090	11.38
cgd8_1770	13.20	cgd7_4880	11.49	cgd6_3920	11.63	cgd6_3920	11.22	cgd2_20	11.36
cgd5_10	13.07	cgd6_3460	11.40	cgd3_3770	11.58	cgd2_20	11.21	cgd7_2430	11.20
cgd3_1570	13.00	cgd3_3770	11.36	cgd6_2330	11.52	cgd6_2090	11.20	cgd2_200	11.09
cgd4_3630	12.88	cgd3_510	11.34	cgd6_10	11.49	cgd7_3120	11.19	cgd4_3620	10.98
cgd2_940	12.83	cgd7_300	11.34	cgd6_3460	11.35	cgd6_2600	11.16	cgd2_940	10.97
cgd4_3620	12.76	cgd6_120	11.29	cgd7_4810	11.31	cgd8_1770	11.07	cgd5_10	10.91
cgd3_410	12.58	cgd4_3550	11.27	cgd8_2930	11.31	cgd7_4810	11.07	cgd4_3630	10.88
cgd6_4910	12.56	cgd8_2930	11.23	cgd6_710	11.26	cgd7_300	11.02	cgd6_200	10.88
cgd3_4150	12.46	cgd6_2330	11.22	cgd6_3790	11.25	cgd7_1730	10.97	cgd6_3460	10.81
cgd1_1580	12.45	cgd2_20	11.19	cgd2_790	11.23	cgd7_2430	10.85	cgd6_3990	10.72
cgd5_2060	12.37	cgd1_640	11.14	cgd2_200	11.22	cgd8_5230	10.77	cgd1_1580	10.66
cgd2_4320	12.34	cgd6_2450	11.06	cgd6_40	11.14	cgd4_3630	10.76	cgd3_4150	10.63
cgd7_360	12.32	cgd7_1730	11.05	cgd2_490	11.11	cgd2_790	10.73	cgd3_410	10.57
cgd6_4860	12.17	cgd3_1570	11.03	cgd8_440	11.09	cgd2_940	10.69	cgd6_4910	10.56
cgd1_880	12.15	cgd6_4460	11.00	cgd7_300	11.07	cgd7_4880	10.66	cgd7_3010	10.46
cgd1_2400	12.10	cgd6_3790	10.91	cgd7_3790	11.07	cgd8_1720	10.65	cgd3_1570	10.40

cgd8_5340	12.04	cgd6_5440	10.90	cgd4_1910	11.07	cgd2_2110	10.62	cgd2_4320	10.37
cgd8_3770	12.04	cgd7_360	10.85	cgd4_2260	11.04	cgd2_200	10.58	cgd7_4810	10.33
cgd6_1430	12.04	cgd5_1210	10.85	cgd5_10	10.94	cgd1_1580	10.55	cgd5_3160	10.33
cgd7_3010	11.99	cgd4_2260	10.80	cgd2_20	10.92	cgd5_10	10.55	cgd5_2060	10.32
cgd5_1470	11.93	cgd2_3110	10.78	cgd7_400	10.90	cgd7_4020	10.54	cgd3_3940	10.30
cgd2_430	11.93	cgd7_3120	10.75	cgd8_1770	10.89	cgd7_400	10.50	cgd6_2450	10.27
cgd3_3340	11.88	cgd8_1720	10.73	cgd7_4880	10.82	cgd4_3620	10.46	cgd8_5340	10.26
cgd8_2050	11.88	cgd8_1160	10.71	cgd6_4470	10.80	cgd6_4910	10.34	cgd6_1430	10.21
cgd4_3400	11.82	cgd8_440	10.69	cgd6_2450	10.77	cgd3_410	10.31	cgd1_2400	10.20
cgd6_4470	11.80	cgd5_1220	10.62	cgd6_3010	10.74	cgd4_680	10.30	cgd7_360	10.18
cgd5_40	11.78	cgd7_1280	10.59	cgd7_1730	10.72	cgd5_3160	10.28	cgd2_790	10.17
cgd7_4790	11.77	cgd7_3790	10.54	cgd5_1210	10.61	cgd3_4150	10.28	cgd6_4860	10.13
cgd5_3160	11.72	cgd5_10	10.49	cgd7_360	10.55	cgd1_590	10.27	cgd7_300	10.12
cgd7_940	11.71	cgd4_1910	10.49	cgd8_1720	10.53	cgd4_3220	10.27	cgd7_3120	10.09
cgd6_780	11.70	cgd6_3920	10.35	cgd6_120	10.52	cgd4_2720	10.23	cgd7_1730	10.09
cgd8_4620	11.68	cgd5_1510	10.34	cgd5_1960	10.50	cgd3_1570	10.21	cgd6_780	10.06

Supplementary Table 5. Raw mRNA reads from time series experiment.

Ct = count; CV = coefficient of variation; S. typhi = *Salmonella typhimurium*; C.p = *Cryptosporidium parvum*; C.h = *Cryptosporidium hominis*; Spor = Sporozoites

**Probe Name**	**Species Name**	**Avg Ct**	**Min Ct**	**Max Ct**	**% CV**	**S. typhi+**	**C.p Spor**	**C.h Spor**	**Cells alone**	**120 h C.p +**	**120 h C. h +**	**96 h C.p +**	**96 h C.h +**	**48 h C.p +**	**48 h C.h +**	**24 h C.p +**	**24 h C.h +**
**ARG1**	*Homo sapiens*	12.17	9	17	20.39	11	17	14	14	11	15	10	9	13	12	9	11
**ARG2**	*Homo sapiens*	175.25	9	327	54.08	249	14	9	194	160	209	274	107	327	196	178	186
**BCL2**	*Homo sapiens*	15	6	22	35.05	20	18	9	20	6	20	13	9	14	12	22	17
**CAMP**	*Homo sapiens*	13.42	9	18	20.71	9	15	15	14	18	10	17	10	12	14	13	14
**CASP1**	*Homo sapiens*	511.58	11	958	59.04	286	17	11	595	534	796	958	294	552	617	794	685
**CASP3**	*Homo sapiens*	388.08	13	687	54.33	370	17	13	417	403	552	687	207	436	461	580	514
**CASP4**	*Homo sapiens*	1163.17	22	1875	53.01	926	30	22	1402	1306	1732	1875	736	1320	1504	1608	1497
**CASP5**	*Homo sapiens*	53	11	89	49.42	37	11	13	45	52	72	89	32	63	83	80	59
**CCL5**	*Homo sapiens*	200.33	10	1128	148.99	1128	10	12	149	96	150	154	46	179	147	174	159
**CD36**	*Homo sapiens*	31.92	7	67	51.59	23	14	7	24	37	54	67	23	33	40	29	32
**CISH**	*Homo sapiens*	27.67	7	40	39.27	24	7	12	20	32	34	40	23	27	34	40	39
**CX3CL1**	*Homo sapiens*	262.58	11	853	82.6	853	13	11	251	251	341	349	102	242	257	274	207
**CXCL8**	*Homo sapiens*	9395	10	44779	124.13	44779	18	10	7634	5502	6776	9273	2944	9103	8488	9462	8751
**DEFA1**	*Homo sapiens*	5.75	3	10	31.57	7	6	6	6	6	5	6	5	3	10	3	6
**DEFB1**	*Homo sapiens*	550	12	1234	65.32	302	17	12	538	648	1056	1234	437	459	576	711	610
**DEFB4B**	*Homo sapiens*	8.5	4	14	42.71	14	11	5	8	5	6	8	5	10	14	12	4
**FAS**	*Homo sapiens*	83.08	5	130	50.25	130	8	5	100	75	89	126	49	104	101	113	97
**FASLG**	*Homo sapiens*	8.17	3	13	34.57	5	7	10	10	5	11	9	3	13	9	8	8
**GUSB**	*Homo sapiens*	811.08	13	1438	54.22	642	16	13	859	874	1159	1438	564	954	944	1151	1119
**HPRT1**	*Homo sapiens*	1805	12	3124	55.32	1549	12	13	1965	1822	2429	3124	1069	2168	2286	2852	2371
**ICAM1**	*Homo sapiens*	2698.58	3	12153	117.14	12153	3	4	2126	1508	2005	2589	796	2855	2626	3037	2681
**IFNG**	*Homo sapiens*	11.25	6	21	39.23	11	21	11	8	9	8	18	6	8	11	14	10
**IL18**	*Homo sapiens*	450.42	12	760	54.17	451	18	12	464	419	553	710	253	581	544	760	640
**IL1B**	*Homo sapiens*	495.25	16	2781	149.87	2781	18	16	351	200	260	258	113	450	427	569	500
**IL1R1**	*Homo sapiens*	274.33	18	511	53.7	176	19	18	290	326	410	511	209	299	321	368	345
**IL33**	*Homo sapiens*	29.25	12	49	36.21	27	17	12	34	29	31	33	15	29	35	49	40

**IL6**	*Homo sapiens*	133	16	694	135.91	694	24	16	81	80	74	102	45	140	105	110	125
**NFKB1**	*Homo sapiens*	184	8	556	75.54	556	16	8	152	139	214	240	103	174	192	212	202
**PTGS2**	*Homo sapiens*	440.42	8	1573	90.88	1573	8	14	332	372	514	626	221	389	398	439	399
**PYCARD**	*Homo sapiens*	1059.92	20	1912	55.94	650	33	20	1119	1210	1550	1912	724	1233	1298	1557	1413
**RELA**	*Homo sapiens*	1721.75	12	2842	52.79	2417	14	12	1771	1841	2443	2842	1044	1961	2002	2154	2160
**RPL19**	*Homo sapiens*	37761.08	24	64785	54.67	31880	24	24	42970	40816	56030	64785	23803	43444	45243	54611	49503
**SDHA**	*Homo sapiens*	791.67	6	1473	55.51	634	13	6	766	889	1052	1473	511	975	1002	1094	1085
**SIRT1**	*Homo sapiens*	110.17	13	182	46.29	130	20	13	110	126	153	182	78	104	147	140	119
**SOCS1**	*Homo sapiens*	186.92	12	336	53.03	236	12	14	186	187	232	336	97	233	190	263	257
**SOCS2**	*Homo sapiens*	57.75	3	113	56.73	113	8	3	47	54	72	88	28	67	75	85	53
**SOCS3**	*Homo sapiens*	637.83	14	1038	51.63	795	16	14	677	719	852	1038	369	780	725	815	854
**SOCS4**	*Homo sapiens*	491.75	10	867	54.35	373	22	10	509	574	712	867	304	568	614	665	683
**STAT6**	*Homo sapiens*	667.83	7	1142	53.67	602	17	7	692	746	1034	1142	440	717	825	901	891
**TRAF2**	*Homo sapiens*	246.58	7	394	53.34	209	12	7	246	279	362	392	153	272	321	394	312
**cgd1_1660**	*C. parvum* Iowa II	41.92	3	389	261.12	10	389	27	8	7	13	10	3	8	9	10	9
**cgd1_2270**	*C. parvum* Iowa II	39.25	5	372	267.22	8	372	20	6	10	6	10	5	14	10	5	5
**cgd1_2400**	*C. parvum* Iowa II	117.92	3	1260	305.1	3	1260	39	12	13	11	12	15	12	11	10	17
**cgd1_2880**	*C. parvum* Iowa II	20.33	10	55	58.84	15	55	29	13	10	19	21	16	13	20	15	18
**cgd1_300**	*C. parvum* Iowa II	142.67	10	1539	308.24	12	1539	30	18	12	17	14	10	12	14	17	17
**cgd1_3810**	*C. parvum* Iowa II	97.08	3	1057	311.51	10	1057	35	4	3	7	12	4	4	12	12	5
**cgd1_640**	*C. parvum* Iowa II	466.08	8	5297	326.44	8	5297	85	16	14	27	47	21	18	21	17	22
**cgd1_880**	*C. parvum* Iowa II	63.5	7	614	273.13	12	614	15	11	9	21	23	13	12	18	7	7
**cgd1_910**	*C. parvum* Iowa II	70.67	7	685	274.05	12	685	40	12	12	10	22	14	9	12	13	7
**cgd2_1070**	*C. parvum* Iowa II	32.5	9	203	165.62	21	203	23	13	16	17	20	9	14	18	19	17
**cgd2_20**	*C. parvum* Iowa II	166.75	10	1785	305.69	21	1785	51	16	13	15	23	11	11	22	23	10
**cgd2_200**	*C. parvum* Iowa II	394	5	4372	318.19	8	4372	180	9	5	23	40	35	11	19	14	12
**cgd2_2200**	*C. parvum* Iowa II	38.58	9	307	219.71	12	307	31	14	12	10	22	15	9	12	10	9

**cgd2_2540**	*C. parvum* Iowa II	31.5	4	286	254.72	4	286	15	6	5	7	13	14	10	5	7	6
**cgd2_3000**	*C. parvum* Iowa II	40.67	1	398	276.96	5	398	19	7	7	9	12	1	12	9	6	3
**cgd2_3070**	*C. parvum* Iowa II	139.08	24	1299	262.72	30	1299	49	31	25	40	28	47	24	45	26	25
**cgd2_350**	*C. parvum* Iowa II	46.75	2	453	273.94	2	453	26	10	9	9	7	5	9	11	12	8
**cgd2_3730**	*C. parvum* Iowa II	121	4	1317	311.39	9	1317	42	9	10	4	19	11	6	9	7	9
**cgd2_430**	*C. parvum* Iowa II	267.17	6	3103	334.27	10	3103	6	7	7	8	21	6	8	11	9	10
**cgd2_4320**	*C. parvum* Iowa II	30.5	9	177	151.85	15	177	19	18	16	18	26	13	9	17	20	18
**cgd2_820**	*C. parvum* Iowa II	103.67	7	1084	297.87	13	1084	34	12	11	12	16	11	16	15	13	7
**cgd3_1300**	*C. parvum* Iowa II	119.25	11	1176	279.22	17	1176	54	15	22	21	29	11	19	27	22	18
**cgd3_1400**	*C. parvum* Iowa II	17.33	8	35	41.11	8	35	23	15	15	11	17	11	18	19	14	22
**cgd3_1570**	*C. parvum* Iowa II	216.5	10	2402	317.91	11	2402	22	13	16	17	30	20	14	26	17	10
**cgd3_1940**	*C. parvum* Iowa II	150.5	16	1543	291.48	21	1543	58	18	16	19	30	18	16	16	30	21
**cgd3_2250**	*C. parvum* Iowa II	49	6	480	277.05	11	480	15	8	9	7	10	9	13	9	6	11
**cgd3_2940**	*C. parvum* Iowa II	18.08	11	56	70.13	11	56	26	15	18	11	12	11	14	14	12	17
**cgd3_330**	*C. parvum* Iowa II	35.58	10	252	191.83	19	252	24	16	11	19	17	13	17	14	10	15
**cgd3_3770**	*C. parvum* Iowa II	375.83	5	4332	331.52	9	4332	57	9	12	10	27	21	9	13	5	6
**cgd3_3790**	*C. parvum* Iowa II	28.67	1	224	215.83	7	224	27	13	8	17	14	5	1	10	11	7
**cgd3_3930**	*C. parvum* Iowa II	157.33	9	1692	307.2	17	1692	30	9	14	18	22	13	21	9	23	20
**cgd3_4150**	*C. parvum* Iowa II	93.5	5	1000	305.42	11	1000	32	5	10	11	16	7	8	5	11	6
**cgd3_4260**	*C. parvum* Iowa II	21.75	3	188	241.02	8	188	4	11	10	5	10	6	4	5	7	3
**cgd3_510**	*C. parvum* Iowa II	180.42	3	1984	314.89	9	1984	51	3	10	16	21	28	8	12	12	11
**cgd3_760**	*C. parvum* Iowa II	65	5	651	284.14	6	651	32	5	9	6	18	7	11	13	11	11

**cgd4_1940**	*C. parvum* Iowa II	21.67	6	136	166.98	14	136	18	6	11	14	13	10	7	7	14	10
**cgd4_2260**	*C. parvum* Iowa II	53.33	6	522	276.96	8	522	29	11	8	10	9	6	10	9	7	11
**cgd4_3080**	*C. parvum* Iowa II	14.67	5	69	118.89	6	69	14	7	9	8	12	5	14	13	13	6
**cgd4_3160**	*C. parvum* Iowa II	30.42	11	98	73.49	31	98	30	11	25	22	27	29	15	20	35	22
**cgd4_3550**	*C. parvum* Iowa II	61.67	6	599	274.56	10	599	26	10	9	15	6	8	15	14	8	20
**cgd4_3620**	*C. parvum* Iowa II	34.08	2	291	237.93	9	291	23	6	7	10	15	11	10	17	8	2
**cgd4_4310**	*C. parvum* Iowa II	63.25	3	596	265.56	18	596	37	3	13	19	12	8	14	13	13	13
**cgd4_4460**	*C. parvum* Iowa II	20.67	10	68	75.65	20	68	27	10	11	17	17	16	12	21	14	15
**cgd5_10**	*C. parvum* Iowa II	166.75	2	1818	312	12	1818	65	4	2	17	15	21	15	17	7	8
**cgd5_1470**	*C. parvum* Iowa II	73.92	4	787	303.84	11	787	16	9	9	6	12	4	7	10	11	5
**cgd5_2370**	*C. parvum* Iowa II	31.25	4	259	229.89	4	259	16	12	13	12	17	6	8	13	5	10
**cgd5_2800**	*C. parvum* Iowa II	49.83	4	475	269.09	6	475	33	11	7	10	8	13	12	12	7	4
**cgd5_3040**	*C. parvum* Iowa II	57.92	3	584	286.25	10	584	27	3	8	12	14	3	8	10	8	8
**cgd5_4590**	*C. parvum* Iowa II	30.33	7	184	160.3	20	184	19	13	17	7	21	13	16	17	25	12
**cgd6_10**	*C. parvum* Iowa II	158.33	7	1736	313.8	20	1736	14	13	18	19	18	13	16	14	12	7
**cgd6_1070**	*C. parvum* Iowa II	10.17	4	41	102.55	6	41	18	8	8	5	5	4	7	4	6	10
**cgd6_1080**	*C. parvum* Iowa II	12.33	4	45	89.91	6	45	7	6	5	11	16	4	17	10	12	9
**cgd6_2170**	*C. parvum* Iowa II	70.58	7	706	283.57	9	706	21	12	10	13	12	7	15	12	21	9
**cgd6_2330**	*C. parvum* Iowa II	19.58	3	109	147.56	11	109	30	11	6	11	11	3	8	10	12	13
**cgd6_2600**	*C. parvum* Iowa II	241.33	6	2760	328.68	7	2760	33	6	9	10	17	12	9	18	8	7
**cgd6_3010**	*C. parvum* Iowa II	35	5	329	264.66	5	329	15	8	6	5	11	9	7	9	10	6
**cgd6_3800**	*C. parvum* Iowa II	35.67	12	147	100.92	31	147	41	18	15	30	33	12	22	24	30	25
**cgd6_3850**	*C. parvum* Iowa II	61.5	3	619	285.71	3	619	31	12	10	7	13	5	10	10	13	5

**cgd6_3920**	*C. parvum* Iowa II	329.5	6	3744	326.37	9	3744	63	6	7	15	31	18	11	23	17	10
**cgd6_3990**	*C. parvum* Iowa II	115.83	6	1265	312.47	8	1265	28	13	8	6	16	8	7	8	15	8
**cgd6_40**	*C. parvum* Iowa II	290.58	14	3229	318.46	25	3229	20	30	17	24	35	14	20	21	30	22
**cgd6_4190**	*C. parvum* Iowa II	26	3	234	252.18	4	234	14	6	7	7	5	7	10	9	6	3
**cgd6_4620**	*C. parvum* Iowa II	66.42	4	676	289.29	9	676	35	7	4	12	13	7	11	9	9	5
**cgd6_4630**	*C. parvum* Iowa II	93.25	2	999	305.98	2	999	30	16	12	6	7	9	8	8	10	12
**cgd6_4860**	*C. parvum* Iowa II	99.92	7	1041	296.76	7	1041	41	9	22	9	16	8	13	14	9	10
**cgd6_4910**	*C. parvum* Iowa II	128.25	7	1385	308.67	11	1385	41	13	10	9	17	13	7	10	11	12
**cgd6_5400**	*C. parvum* Iowa II	68	1	758	319.6	2	758	14	5	3	7	9	1	4	6	6	1
**cgd6_5410**	*C. parvum* Iowa II	461.83	10	5223	324.72	10	5223	114	18	19	17	48	28	19	23	12	11
**cgd7_120**	*C. parvum* Iowa II	43.83	9	353	222.91	17	353	41	13	12	13	9	14	15	13	11	15
**cgd7_130**	*C. parvum* Iowa II	71.83	17	618	239.52	24	618	33	18	17	19	24	19	23	26	19	22
**cgd7_1460**	*C. parvum* Iowa II	70.33	8	713	287.83	13	713	25	9	10	15	9	8	13	10	10	9
**cgd7_1830**	*C. parvum* Iowa II	8.75	4	37	106.56	5	37	14	7	5	4	7	4	5	5	8	4
**cgd7_2110**	*C. parvum* Iowa II	67.42	8	672	282.52	11	672	27	8	10	12	10	9	18	9	12	11
**cgd7_2250**	*C. parvum* Iowa II	21	9	93	109.76	15	93	25	11	10	18	14	9	15	14	13	15
**cgd7_2280**	*C. parvum* Iowa II	59.75	6	582	275.38	11	582	24	8	14	8	17	11	9	15	12	6
**cgd7_2300**	*C. parvum* Iowa II	383.33	8	4389	329.1	8	4389	60	12	14	10	44	11	16	13	13	10
**cgd7_2430**	*C. parvum* Iowa II	269.42	7	2988	317.82	15	2988	66	20	14	13	27	24	17	20	22	7
**cgd7_2540**	*C. parvum* Iowa II	76.17	5	796	297.76	6	796	31	11	11	10	7	6	12	5	12	7
**cgd7_3010**	*C. parvum* Iowa II	43.58	3	399	257.05	11	399	13	8	6	10	23	13	12	3	12	13
**cgd7_3020**	*C. parvum* Iowa II	26.17	8	158	159.64	15	158	23	12	9	8	10	13	18	15	21	12

**cgd7_3240**	*C. parvum* Iowa II	39.33	8	340	241.11	8	340	27	8	10	8	17	10	11	12	9	12
**cgd7_3250**	*C. parvum* Iowa II	99.33	2	1064	305.92	8	1064	33	2	6	8	14	10	14	10	13	10
**cgd7_4050**	*C. parvum* Iowa II	71.25	8	717	285.55	12	717	31	13	10	9	10	8	8	15	11	11
**cgd7_480**	*C. parvum* Iowa II	326.83	5	3696	324.71	7	3696	86	6	5	17	27	32	9	21	7	9
**cgd7_700**	*C. parvum* Iowa II	12.67	3	49	96.06	14	49	15	11	6	6	6	3	6	8	15	13
**cgd7_790**	*C. parvum* Iowa II	45.25	2	441	275.54	9	441	13	9	6	12	14	9	6	14	8	2
**cgd8_1840**	*C. parvum* Iowa II	62.5	17	448	194.61	29	448	41	23	17	31	23	19	27	39	32	21
**cgd8_2340**	*C. parvum* Iowa II	36	3	291	223.35	17	291	19	15	3	13	16	11	11	12	13	11
**cgd8_3230**	*C. parvum* Iowa II	153.33	3	1691	315.91	6	1691	49	9	3	10	20	6	13	14	11	8
**cgd8_3390**	*C. parvum* Iowa II	43.5	6	366	233.96	23	366	29	13	7	9	15	13	6	14	10	17
**cgd8_3460**	*C. parvum* Iowa II	10.5	6	18	34.82	11	18	6	7	8	12	12	13	9	15	8	7
**cgd8_3480**	*C. parvum* Iowa II	73.17	3	785	306.43	7	785	17	5	8	8	8	3	13	10	10	4
**cgd8_3520**	*C. parvum* Iowa II	600	8	6855	328.35	11	6855	132	8	10	26	53	27	25	27	14	12
**cgd8_3770**	*C. parvum* Iowa II	65.92	10	608	259.11	14	608	30	19	17	12	20	12	10	18	12	19
**cgd8_4050**	*C. parvum* Iowa II	102.42	8	1005	277.63	24	1005	35	25	20	13	20	11	8	24	27	17
**cgd8_4220**	*C. parvum* Iowa II	56.75	17	419	201.18	20	419	34	25	25	25	28	21	17	26	18	23
**cgd8_4320**	*C. parvum* Iowa II	222.17	4	2447	315.47	4	2447	72	21	8	18	27	16	14	14	12	13
**cgd8_4500**	*C. parvum* Iowa II	21.33	5	133	167.46	15	133	28	6	5	13	6	5	10	13	11	11
**cgd8_60**	*C. parvum* Iowa II	101	4	1058	298.43	15	1058	15	12	15	4	21	8	20	20	12	12
**cgd_2_140**	*C. parvum* Iowa II	192.08	5	2186	326.93	5	2186	35	8	7	10	9	8	11	5	12	9
**cgd2_3270**	*C. parvum* Iowa II	19.67	3	118	164.44	6	118	39	10	10	9	6	7	11	12	3	5
**cgd5_3160**	*C. parvum* Iowa II	38.25	6	326	237.2	13	326	24	12	11	6	9	13	8	15	10	12
**cgd6_2090**	*C. parvum* Iowa II	11.83	1	58	133.95	7	58	26	8	5	5	11	6	7	5	1	3

**cgd6_4270**	*C. parvum* Iowa II	27.08	12	159	153.65	16	159	21	15	12	17	15	13	16	15	14	12
